# Microfluidic QCM enables ultrahigh *Q*-factor: a new paradigm for in-liquid gravimetric sensing

**DOI:** 10.1038/s41378-024-00732-2

**Published:** 2024-08-26

**Authors:** Yicheng Zhao, Zehra Parlak, Wenjun Yu, Daniel French, Wilkins Aquino, Stefan Zauscher

**Affiliations:** 1 Thomas Lord Department of Mechanical Engineering and Materials Science, Duke University, Durham, NC USA; 2 Qatch Technologies, LLC., Durham, NC USA; 3 Department of Civil and Environmental Engineering, Duke University, Durham, NC USA

**Keywords:** Engineering, Nanofluidics

## Abstract

Acoustic gravimetric biosensors attract attention due to their simplicity, robustness, and low cost. However, a prevailing challenge in these sensors is dissipation which manifests in a low quality factor (*Q*-factor), which limits their sensitivity and accuracy. To mitigate dissipation of acoustic sensors in liquid environments we introduce an innovative approach in which we combine microfluidic channels with gravimetric sensors. To implement this novel paradigm we chose the quartz crystal microbalance (QCM) as our model system, owing to its wide applicability in biosensing and the relevance of its operating principles to other types of acoustic sensors. We postulate that the crucial determinant for enhancing performance lies in the ratio between the width of the microfluidic channels and the wavelength of the pressure wave generated by the oscillating channel side walls driven by the QCM. Our hypothesis is supported by finite element analysis (FEA) and dimensional studies, which revealed two key factors that affect device performance: (1) the ratio of the channel width to the pressure wavelength ($W/{\lambda }_{{\rm {p}}}$) and (2) the ratio of the channel height to the shear evanescent wavelength ($H/{\lambda }_{{\rm {s}}}$). To validate our hypothesis, we fabricated a microfluidic QCM (µ-QCM) and demonstrated a remarkable 10-fold improvement in its dissipation when compared to conventional QCM. The novel microfluidic approach offers several additional advantages, such as direct data interpretation, reduced volume requirement for sample liquids, and simplified temperature control, augmenting the sensor’s overall performance. By fostering increased sensitivity, accuracy, and ease of operation, our novel paradigm unlocks new possibilities for advancing gravimetric technologies, potentially for biosensing applications.

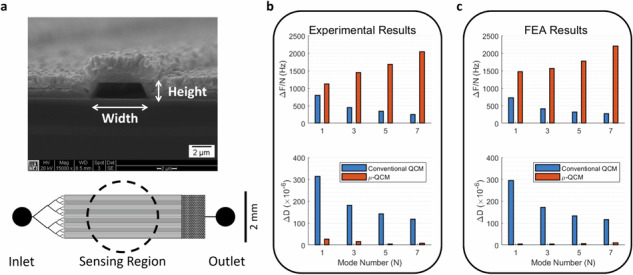

## Introduction

Biosensing plays a crucial role in detecting and quantifying biological signals^1–4^ or molecules^5–7^. Its applications range from improving healthcare^8–10^ to monitoring environmental pollutants^11–13^. As an essential tool in biology, medicine, and environmental science, biosensing remains an area of continuous research and development^14–18^.

Biosensors employ various transduction principles, including electrical^19–21^, optical^9,22,23^, and acoustic methods^24–26^. Among these, acoustic gravimetric biosensors stand out for their simplicity, robustness, and low cost, making them attractive for a wide range of applications, particularly in point-of-care (POC) settings^27,28^.

Despite their advantages, acoustic biosensors often face a common drawback—a low-quality factor (*Q*-factor)^25^ in liquid measurements. The *Q*-factor ($Q$), which measures energy loss (or dissipation, $D=1/Q$) in an acoustic system, significantly impacts the sensitivity and accuracy of acoustic biosensors^29,30^. For out-of-plane mode acoustic biosensors, low *Q*-factors mainly result from acoustic radiation energy loss. Hence, in-plane mode operation is preferred as it avoids direct acoustic energy emission into the surrounding environment. Nevertheless, even in-plane acoustic biosensors encounter energy dissipation due to friction within the shear evanescent boundary layer^25^.

Over the years, researchers have explored several methods to enhance the *Q*-factors of acoustic biosensors. These methods include using wave interference^31^, isolating sensors from their surroundings^26^, and implementing meta structures to trap acoustic energy^32^. However, these efforts have only partially addressed the issue of low *Q*-factors, without fully resolving the underlying challenge. In this context, we demonstrate an innovative acoustic biosensor design that effectively eliminates dissipation in the liquid phase, leading to a significant improvement in the *Q*-factor. By tackling this fundamental issue, our design opens new possibilities for advancing the performance and capabilities of acoustic biosensors for diverse applications.

We implemented our paradigm using a quartz crystal microbalance (QCM) as the model system. QCMs are thickness shear mode (TSM) resonators capable of detecting surface-bound mass and the associated energy loss by measuring resonance frequency shifts ($\Delta f$) and dissipation shifts ($\Delta D$), respectively^25,33–36^. The selection of a QCM as a model system is justified by its simplicity and widespread application^37,38^, low production cost^29^, and the relevance of its operating principles to other acoustic gravimetric biosensors, including surface acoustic wave (SAW) sensors^39^, film bulk acoustic resonator (FBAR) sensors^26^, and other bulk acoustic wave (BAW) sensors.

Our approach incorporates rigid microfluidic channels on conventional QCM crystals, leading to the creation of a new device we termed microfluidic QCM (µ-QCM). Through a comprehensive combination of simulations, theoretical studies, and experiments, we demonstrate a 10-fold decrease in dissipation shift ($\Delta D$) induced by liquid loading. In addition to the significant decrease in dissipation, the µ-QCM offers several other advantages. These include direct data interpretation, significantly reduced sample volume requirements, and less stringent temperature control, all of which render the µ-QCM potentially attractive for POC sensing applications.

Our research also aimed at gaining a deeper understanding of the physics underlying µ-QCM operation. For the µ-QCM to exhibit low dissipation (i.e., high *Q*-factor) in liquid, we found that the width of the microfluidic channels has to be significantly smaller than the pressure wavelength across the channel width, while the channel height does not necessarily need to be smaller than the penetration length of the evanescent shear wave. Our findings provide valuable insights into the design and performance of other acoustic biosensors and thus contribute more broadly to advancing acoustic gravimetric biosensor technologies and their practical implementations.

## Results

### Device design

To suppress dissipation, we confined the sample liquid in many parallel rigid microfluidic channels, on top of the QCM crystal sensor. These small channels (2 µm × 10 µm cross-sectional dimension) are oriented *perpendicular* to the shearing direction of the QCM crystal, which generates pressure waves in the sample liquid across the microfluidic channels. We note that non-uniform shear displacement-inducing pressure waves in QCM are well-known^40^. To minimize dissipation ($D$) we kept the channel width ($W$) to less than one-quarter of the pressure wavelength (${\lambda }_{{\rm {p}}}$) in the sample liquid.

**Fig. 1:**
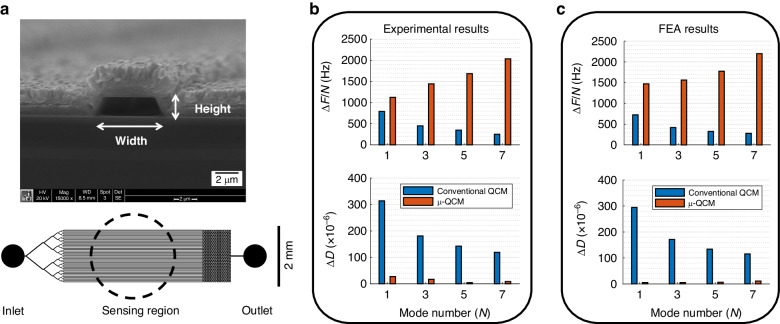
Design and characteristics of a µ-QCM. **a** Schematic showing the microfluidic channel system (with ~100 channels) placed over the working area (dashed circle) of a QCM sensor. Liquid is driven into the channels from left to right through capillary action. The SEM micrograph shows the cross-section of an individual channel. Normalized frequency and dissipation shift of the conventional and µ-QCM plotted as a function of mode number for **b** experiments and **c** FEA simulations

Commonly employed quartz crystals in conventional QCM have a thickness of ~330 µm, resulting in a fundamental frequency of ~5 MHz^36^. Since these sensors can be operated at higher harmonic frequencies, we designed our µ-QCM to operate at least up to the 7th overtone. The target frequency for our design was, therefore, 35 MHz, which corresponds to a pressure wavelength of ~42 µm in water^41^. We thus limited the width of the microfluidic channels to 10 µm. Furthermore, to avoid mass overloading of the sensor, we limited the channel height to 2 µm, which is still much larger than the evanescent shear wavelength (${\lambda }_{{\rm {s}}}\approx 250\,{{\rm {nm}}}$). Finally, to ensure sufficient sensitivity, we placed ~100 microfluidic channels, spaced 20 µm apart, centrally over a width of 3 mm on the active area of the sensor (indicated by the dashed line in Fig. 1a).

In the current design, the liquid is entrained into the channels solely through capillary action, and complete channel filling is key to the repeatability of µ-QCM measurements. We found, however, that without a suitably diverging entrance channel system and a wicking outlet design, the liquid front in each channel does not reach the channel termini simultaneously. In this situation, the liquid that reaches the channel end first will spread and impede the outflow from adjacent channels. To ameliorate this problem and maintain a steady and bubble-free flow, we implemented (i) a diverging approach flow channel system to connect our inlet to the 100 parallel channels and (ii) micro-pillows, evenly distributed between the outlet and the end of the parallel channels, to ensure bubble-free merging (for further detail see schematic in Fig. S21). Both design features were inspired by the tree-line design for capillary pumping^42–44^.

To minimize energy loss, the microfluidic channels must be stiff, i.e., have a high Young’s modulus, and be lightweight, as excessive mass in the channel walls can critically damp and overload the QCM crystal. Given these constraints, we chose to fabricate the microfluidic channels from aluminum due to its high modulus-to-density ratio. Furthermore, to enable surface modification of the inner surfaces of the channels, we introduced a thin (~40 nm), conformal gold coating in the fabrication process (see also Fig. 7, see the “Methods” section).

### µ-QCM improves dissipation by 10-fold

Upon admission of DI water to the sensor surface, we observed a substantial dissipation shift ($\Delta D$) for the conventional QCM (about one order of magnitude larger than that of the µ-QCM in Fig. 1b). For conventional QCM, the acoustic energy transfer into the liquid medium occurs *via* an evanescent shear wave. Energy is lost throughout the evanescent shear boundary layer in the form of friction and heat conduction between liquid molecules^30,35^. In contrast, the dissipation shift ($\Delta D$) of the µ-QCM remains small after admitting DI water to the microfluidic channels.

While this observation could be interpreted as the µ-QCM containing a much smaller liquid volume which would not generate significant dissipation and affect the *Q*-factor, we found that the µ-QCM had a normalized resonance frequency shift (measured at the 7th mode) that is 5 times larger than that of a conventional QCM (Fig. 1b). A larger normalized resonance frequency shift indicates that a greater liquid mass is coupled to the sensor^34^. We thus conclude that the µ-QCM couples more DI water while generating significantly less dissipation, which dramatically increases the Q-factor. The underlying physics of these phenomena will be further explained in the finite element analysis (FEA) section, below.

For a conventional QCM, the normalized frequency, calculated as the ratio of the overtone frequency shift to the overtone number ($\Delta F/n$), decreases as the overtone number increases (Fig. 1b). The normalized frequency shift is a measure of the surface-coupled mass ($\Delta m$) to the crystal surface, as established by the Sauerbrey equation^34^ (Eq. (1)). Constants in this equation are the active surface region of the QCM ($A$), the resonance frequency (*f*_*0*_), the shear modulus of AT-cut quartz $({\eta }_{{\rm {q}}})$, and the density of quartz (${\rho }_{{\rm {q}}}$).

1 $$\Delta f=-\frac{2{f}_{0}^{2}}{A\sqrt{{\eta }_{{\rm {q}}}{\rho }_{{\rm {q}}}}}\Delta m$$

Considering the relationship between wavelength and frequency, $\lambda =c/f$, the Sauerbrey equation shows that the wavelength decreases with increasing frequency. The same principle is true for the penetration length of the shear evanescent wave. As the shear evanescent wave extends less into the liquid, less mass is coupled to the crystal surface, which results in a decrease in normalized frequency (Fig. 1b).

However, this trend is inversed for the µ-QCM, where the normalized frequency shift increases with increasing overtone number. While we have observed this effect in our experiments and simulations (Fig. 1b, c), a quantitative explanation is still elusive. Nonetheless, this phenomenon can be harnessed to increase the sensitivity of acoustic sensors.

We note that the microfluidic channels in all our µ-QCMs are oriented *perpendicular* to the shearing direction of the QCM crystal. When the orientation of the liquid-filled microfluidic channels is changed from perpendicular to parallel to the shearing direction, a high dissipation, similar to that of conventional QCM, is observed (Fig. S22). This behavior arises because the shearing motion of the QCM crystal no longer induces pressure waves across the width of the microfluidic channels, thus not suppressing dissipation. However, evanescent shear waves at the channels’ inner walls now persist which results in behavior similar to that of a conventional QCM. This arises because the liquid in the far field does not oscillate, and a boundary layer with a continuously decreasing velocity profile is formed. These velocity differences cause energy dissipation through friction of liquid molecules.

**Fig. 2:**
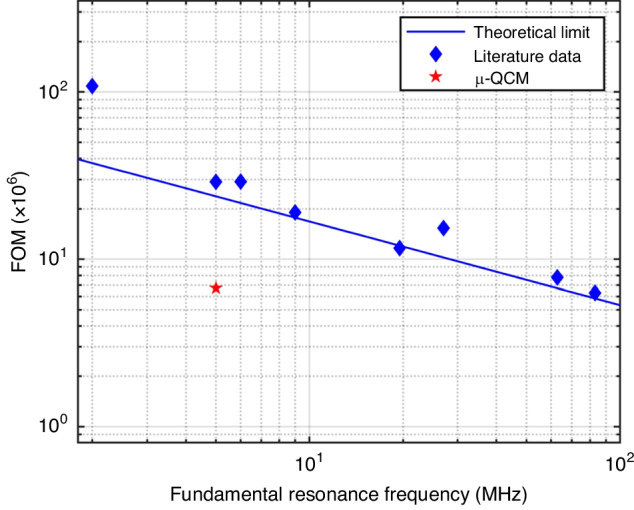
FOM for conventional QCMs and µ-QCM. The theoretical limit of the FOM for AT-cut QCM operating in water is determined by liquid damping (solid blue line). Literature data^45–47^ (blue rhombus) show that conventional QCMs with different thicknesses perform similar to or worse than this FOM limit. The µ-QCM (red star), on the other hand, significantly surpasses the theoretical FOM limit

The mass resolution (i.e., noise equivalent mass resolution) of BAW mass sensors serves as a figure of merit (FOM). According to the definition, ${{\rm {FOM}}}={f}_{0}/(Q\times {s})$, where ${f}_{0}$ is the fundamental resonance frequency, $Q$ is the *Q*-factor, and $s$ is the mass sensitivity (for instance, $s=2.26\times {10}^{-6}{f}_{0}^{2}{{\rm {c}{m}}}^{2}\cdot {{\rm {Hz}/g}}$ for AT-cut QCM)^30^. For BAW mass sensors employing the same waveform and fundamental frequency, the FOM is entirely determined by $Q$. To demonstrate how the design of the µ-QCM can effectively enhance the FOM (by reducing dissipation, i.e., improving the *Q*-factor), we conducted a literature review and plotted the FOM results of conventional QCMs (from literature^45–47^) against that of our µ-QCM in Fig. 2. To ensure the consistency of the data, we specifically selected research that applied conditions like ours, i.e., where QCM devices were used in aqueous environments and had AT-cut crystal orientations. Furthermore, to ensure the reliability of the data, we selected QCM data from experiments over a range of fundamental frequencies. Figure 2 shows that the µ-QCM, by reducing dissipation and thus improving the *Q*-factor, significantly surpasses the theoretical limit of conventional QCMs in liquid.

### µ-QCM responds to only density (mass) change

For a conventional QCM operating in liquid, the frequency shift of the crystal can be predicted by the extended Sauerbrey equation^33^ (Eq. (2)):

2 $$\Delta f=-{f}_{0}^{\frac{3}{2}}\sqrt{{\mu }_{{{l}}}{\rho }_{{{l}}}/\pi {\eta }_{{\rm {q}}}{\rho }_{{\rm {q}}}}$$

which posits a linear relationship between frequency shift and the square root of liquid viscosity multiplied by liquid density ($-\sqrt{{\mu }_{{{l}}}{\rho }_{{{l}}}}$).

On the other hand, the µ-QCM only responds to liquid density (or mass), because it is insensitive to dissipation losses associated with liquid viscosity. We thus expect the µ-QCM to behave according to predictions of the original Sauerbrey equation (Eq. (1)), where the frequency changes linearly with a change in surface-coupled mass.

**Fig. 3:**
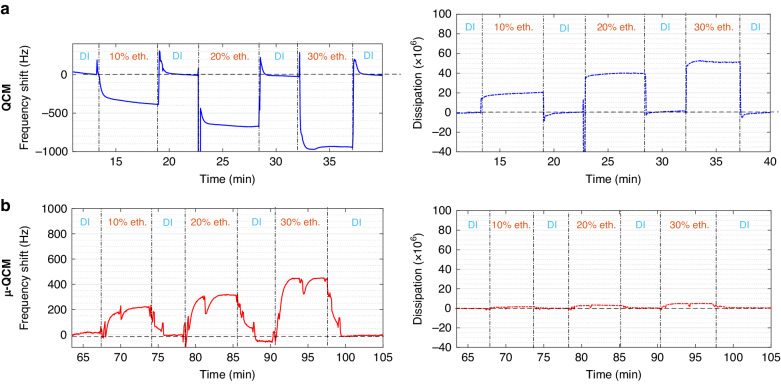
Sensor response to a range of ethanol-water mixtures. **a** The normalized frequency shift for the conventional QCM decreases with increasing wt% of ethanol, as it is largely driven by the increasing viscosity of the mixture. **b** In contrast, the normalized frequency shift of the µ-QCM increases because it only depends on the decreasing density of the mixture. In addition, while dissipation increases with increasing viscosity for the conventional QCM, it is negligible for the µ-QCM

To explore whether the original Sauerbrey equation (Eq. (1)) describes the frequency shift of the µ-QCM, we used ethanol–water mixtures, where density and viscosity as a function of temperature and composition are well known. With increasing weight percent of ethanol (up to 30 wt% ethanol), the viscosity of these mixtures increases significantly while the density decreases slightly. For example, 30 wt% ethanol is expected to have 129.4% higher viscosity and only 4.1% lower density than DI water. For the conventional QCM, we thus expect a square root dependence of the frequency shift on the product of viscosity and density, resulting in a frequency decrease with increasing weight percent of ethanol. In contrast, for the µ-QCM we expect that changes in liquid viscosity have no effect. Thus, we expect that the µ-QCM readily detects the small changes in the liquid density of the water-ethanol mixtures. A decrease in liquid density will cause an increase in the resonance frequency because it entails less mass being coupled to the sensor surface. These predictions are born out in the experimental data shown in Fig. 3 and illustrate the remarkably different behaviors of conventional and µ-QCM, and the latter’s direct mass-sensing capability. The spikes in the data visible in Fig. 3b for the µ-QCM are due to intermittent pipetting of liquid onto the inlet channel. This was done as a preventive measure to mitigate the introduction of bubbles into the channels due to liquid evaporation at the liquid inlet and outlet. The detailed experimental procedure is schematically shown in Fig. S21.

### FEA model of µ-QCM is experimentally validated

We validated our FEA models for both the conventional QCM and the µ-QCM by comparing the model predictions with experiments using DI water as a test liquid. In Fig. 1b, c we compare FEA predictions with experiments in terms of the normalized resonance frequency shifts and their corresponding dissipation shifts for modes 1–7.

Our FEA model for the conventional QCM accurately predicts the experimental results within a 5% error for all modes (Fig. 1b, c). The model also predicts the experimentally observed decrease of the resonance frequency shifts and the corresponding decrease in dissipation shifts with increasing mode number. As discussed above, this behavior arises from the decrease in the shear evanescent wavelength with increasing mode number.

For the µ-QCM, the FEA-predicted normalized frequency shifts are also consistent with our experimental results. However, the corresponding dissipation shifts only agree with experimental results for modes 5 and 7. As discussed next, we attribute this discrepancy to limitations inherent in the assumptions made in the FEA model.

In any QCM, the oscillatory shearing displacement is strongest at the center of the sensor and decays towards the edges. Additionally, as the mode number increases, the shearing displacement becomes more localized around the center region of the sensor. However, in our µ-QCM experiments, a small droplet of sample liquid is deposited on the fluid inlet, which is located outside the active area, close to the outer edge of the quartz crystal (Fig. S20). The liquid is driven into the microchannels from this inlet entirely by capillary action. At lower modes, the presence of this liquid droplet is detected by the sensor as added mass and causes a non-negligible dissipation shift in addition to that generated from the center (active) region of the sensor. The corresponding normalized frequency shift is, however, negligible in comparison to that generated by the presence of liquid in the microfluidic channels in the center region. To mitigate such unwanted contributions from mass loading at the device periphery one can, in future designs, locate the liquid inlet and outlet even further away from the center sensing region of the µ-QCM.

To achieve a computationally efficient FEA model for the µ-QCM, we represented the device by a single repeating unit located in the center region of the sensor. Inherently, however, this model does not capture information from the outer edges of the device. This explains why our simulation results agree more accurately with experimental results at higher modes.

### A pressure wave generated by the channel side walls enables low dissipation

**Fig. 4:**
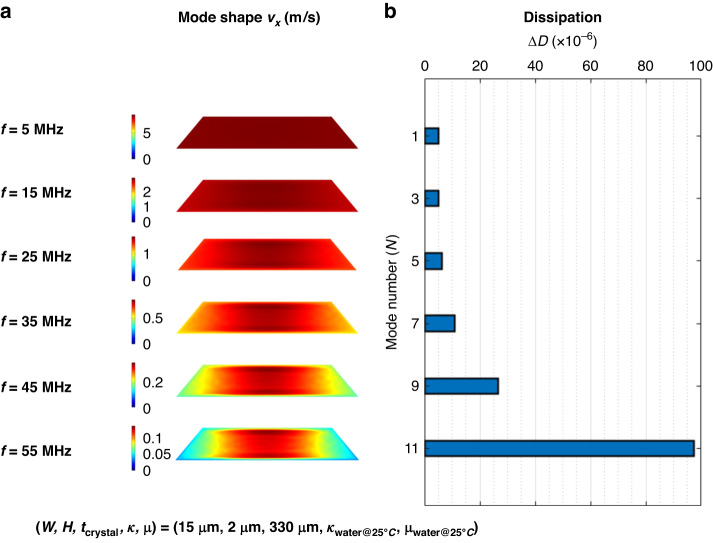
Mode shape and dissipation. **a** Mode shape gradually starts to show variations with increasing mode number. **b** Dissipation increases sharply after the 9th mode

To identify the key factors that contribute to the low dissipation (high *Q*-factor) of the µ-QCM, we conducted a series of parametric FEA studies. Our FEA analysis revealed that the ratio of the width of a microfluidic channel to the wavelength of the pressure wave (i.e., $W/{\lambda }_{{\rm {p}}}$) is the factor that affects dissipation the most. Specifically, we found that as long as this ratio is lower than about 0.2, dissipation in the µ-QCM is small ($\le 20\times {10}^{-6}$). In this case, assuming that the channels are rigidly coupled to the crystal surface and that non-slip boundary conditions are valid at the channel walls, there is no variation in velocity between the liquid at the center of a channel and that at the four-channel wall boundaries. This results in the absence of dissipation caused by frictional forces within the liquid. By observing changes in mode shape and dissipation with increasing mode number (*n*) for a µ-QCM filled with DI water (Fig. 4), we found that once the 5th mode was exceeded, sufficiently large velocity differences of the liquid in the channel occur (Fig. 4a) and give rise to dissipation (Fig. 4b).

**Fig. 5:**
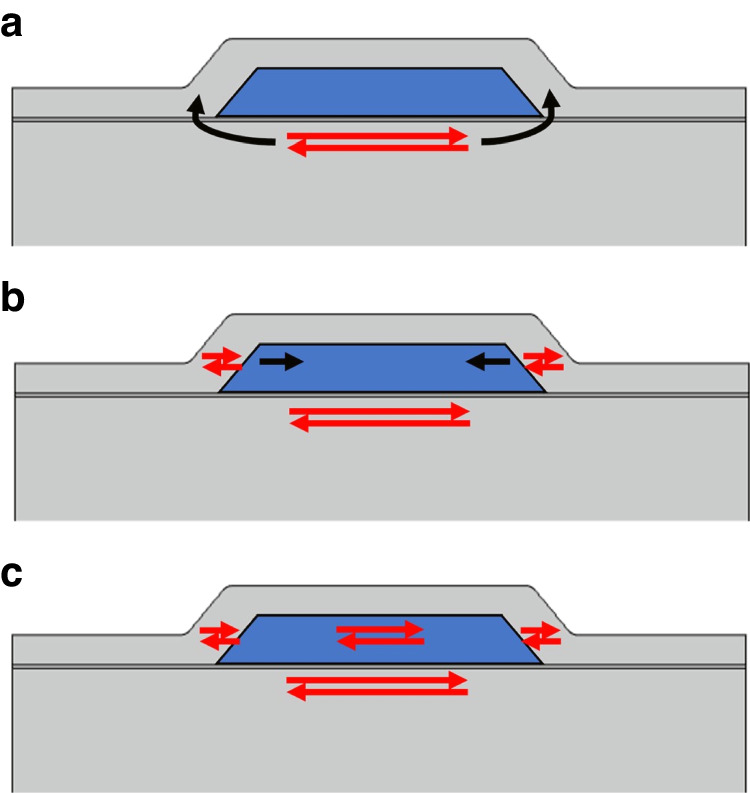
Pressure wave generation process. **a** A quartz crystal resonator’s shearing motion is transmitted to the side walls, **b** the side walls push on the liquid inside the channels, and **c** a pressure wave is set up across the liquid-filled channel

To understand this behavior more clearly, we need to consider the effect of the resonance frequency on the generation of the pressure wavelength (Fig. 5). The pressure wave is excited by the channel side walls which are rigidly coupled to the crystal surface. With increasing mode number, the resonance frequency increases which entails a decrease of the pressure wavelength in the liquid across a channel. This is shown in the spatially resolved velocity profiles of Fig. 4a, where the channel width remains constant as the mode number increases, which results in an increasing $(W/{\lambda }_{{\rm {p}}})$ ratio. When this ratio is >~0.2 in our FEA simulations, the channel accommodates ~20% of the pressure wave’s wavelength, which causes noticeable velocity variations (and thus friction) in the liquid across the channel width.

Dissipation caused by liquid friction can be observed in our simulations of a conventional QCM at all modes and in a µ-QCM above the 5th mode, in the form of an evanescent acoustic shear wave in the liquid. This is illustrated in Fig. S11 for the conventional QCM. At a fundamental frequency of 5 MHz the evanescent acoustic shear wave has a wavelength of 250 nm^48,49^. As the crystal surface oscillates, the liquid in proximity to the surface oscillates at the same velocity and phase as the surface, due to the no-slip boundary condition there. However, the liquid in the far field does not oscillate. As a result, a boundary layer with a continuously decreasing velocity profile is formed. These velocity differences cause energy dissipation through friction of liquid molecules.

For the µ-QCM above the 5th mode, the ($W/{\lambda }_{{\rm {p}}}$) ratio increases with increasing mode number and approaches a value of ~0.2. This means that the channel width accommodates ~0.2 of the pressure wave’s wavelength. In this situation, significant velocity variations occur in the liquid and result in a non-negligible velocity difference between the liquid at the center of the channel and at the channel walls. Like in the conventional QCM, these velocity differences cause energy dissipation through friction of liquid molecules.

In the FEA simulations, we manipulated the mode number, as its effects can be experimentally verified without changing the channel dimensions. However, simulations also allow us to alter parameters that are more difficult to manipulate experimentally, thus providing deeper insight into the underlying physics of the µ-QCM. For example, varying the channel width in our simulations not only modifies the ($W/{\lambda }_{{\rm {p}}}$) ratio but also influences the resonance frequency response of the sensor due to its effect on the amount of liquid coupled in the microfluidic channels. Increasing channel width would also increase the resonance frequency (see Eq. (1)). Alternatively, the pressure wave wavelength can be altered by changing the bulk compressive modulus $(\kappa )$ of the sample liquid in the simulations. The pressure wave wavelength $({\lambda }_{{\rm {p}}})$ is described by Eq. (3)^41,48,49^ (we note that the shear modulus ($\mu$) was neglected from the final expression since it is much smaller than the bulk compressive modulus),

3 $${{\rm{\lambda }}}_{{\rm{p}}}=\frac{{{c}}_{{\rm {p}}}}{{{f}}_{0}}=\sqrt{\frac{{\rm{\kappa }}+\frac{4}{3}\mu }{{\rho }_{{\rm {l}}}{f}_{0}^{2}}}\approx \sqrt{\kappa /{\rho }_{{\rm {l}}}{f}_{0}^{2}}$$

**Fig. 6:**
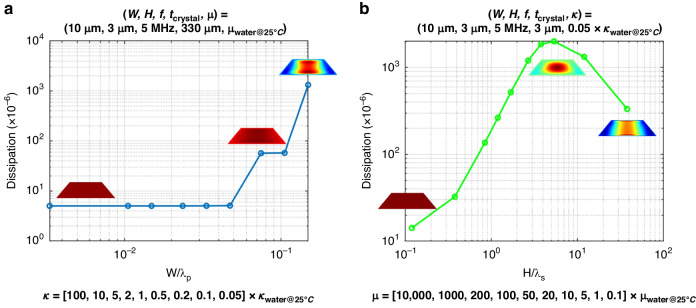
Dissipation responds to $W/{\lambda }_{{\rm {p}}}$ and $H/{\lambda }_{{\rm {s}}}$, demonstrating the effects of pressure waves and shear evanescent waves, respectively. **a** The viscosity is kept constant and the same as that of water at 25 °C. The channel width is set to 10 µm and height is set to 3 µm. Dissipation decreases asymptotically and mode shape becomes uniform with decreasing $W/{\lambda }_{{\rm {p}}}$. **b** The bulk compressive modulus is kept at 0.05 times that of water at 25 °C to ensure that the pressure wave effects do not dominate over the shear evanescent wave effects. Channel width is set to 10 µm and height is set to 3 µm. Dissipation first increases and then asymptotically decreases with decreasing $H/{\lambda }_{{\rm {s}}}$

In Fig. 6 we plot the dissipation as a function of $W/{\lambda }_{{\rm {p}}}$, accompanied by the velocity profile mode shape in the X direction for different $W/{\lambda }_{p}$ values. The channel width is 10 µm and the height is 3 µm. The simulation results in Fig. 6a show that an increase in $W/{\lambda }_{\rm {{p}}}$ (beyond certain value) increases dissipation. The mode shape change provides insight into how pressure waves generated by the side walls can effectively suppress the evanescent acoustic shear wave. Thus, we conclude that with a decreasing ratio of channel width to pressure wavelength ($W/{\lambda }_{{\rm {p}}}$), the shear evanescent wave is suppressed and dissipation is minimized.

### Dimensional analysis helps to identify underlying physics

Through dimensional analysis, we identified two key dimensionless parameters: (1) the ratio of channel width to pressure wave wavelength ($W/{\lambda }_{{\rm {p}}}$) and (2) the ratio of channel height to shear evanescent wavelength ($H/{\lambda }_{{\rm {s}}}$). Above, we already demonstrated the impact of the first ratio on the performance of the µ-QCM. Here, we evaluate the influence of the second dimensionless parameter.

The shear wavelength increases with increasing viscosity as indicated by Eq. (4)^41,48,49^,

4 $${{\rm{\lambda }}}_{{\rm{s}}}=\sqrt{{\mu }_{{\rm{l}}}/\pi {f}_{0}{\rho }_{{\rm{l}}}}$$

In Fig. 6b we plot the dissipation as a function of $H/{\lambda }_{{\rm{s}}}$ (The bulk compressive modulus is kept at 0.05 times of water at 25 °C to ensure that the pressure wave effects do not dominate over the shear evanescent effects). As before, the plot is accompanied by the velocity profile mode shape (from FEA) in the *X* direction for different $H/{\lambda }_{{\rm{s}}}$ values. At first glance, one would expect that an increase in $H/{\lambda }_{{\rm{s}}}$ would entail an increase in dissipation due to increased friction (from higher viscosity) in the system. However, this is not observed for the µ-QCM. As seen in Fig. 6b, dissipation initially increases but then gradually decreases. This suggests the presence of two competing factors, the first being the friction introduced by the increased viscosity, and the second being an increase in the shear evanescent wave wavelength (${\lambda }_{{\rm{s}}}$) to a value much greater than the channel height (*H*). The mode shape changes with increasing $H/{\lambda }_{{\rm{s}}}$ provides insight into how shear wavelength evanescent wave could be used to cancel dissipation in µ-QCM.

**Fig. 7:**
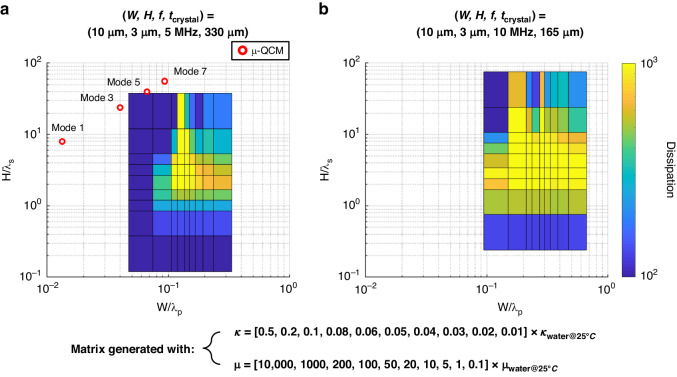
Dimensional design map. Dissipation is generally minimized with increasing $H/{\lambda }_{{\rm{s}}}$ and with selecting a $W/{\lambda }_{{\rm {p}}}$ outside a critical region (yellow highlight). When increasing the fundamental resonance frequency, e.g., through crystal thinning from **a** 5 MHz to **b** 10 MHz, the limits become more stringent for *W* and *H*, because ${\lambda }_{{\rm {p}}}$ and ${\lambda }_{{\rm {s}}}$ are shorter at higher frequencies. The red circles in the figure represent various modes of the µ-QCM used in the experiment

We also combined dimensional analysis with comprehensive numerical simulations (Fig. 7) to extend the applicability of our findings to other acoustic biosensors. The simulations were conducted using our FEA model of the µ-QCM to quantify the dissipation as a function of the two dimensionless groups ($W/{\lambda }_{{\rm{p}}}$ and $H/{\lambda }_{{\rm{s}}}$) at two different fundamental frequencies (5 and 10 MHz).

In general, regardless of the fundamental frequency, dissipation increases with increasing value of either of these two dimensionless groups. This occurs when the critical dimension ($W$ or $H$) increases relative to the wavelength (${\lambda }_{{\rm {p}}}$ or ${\lambda }_{{\rm {s}}}$). However, when the critical dimension is smaller than about one-quarter of the wavelength, then it is too small to accommodate fluid with a significant velocity difference in that dimension. The wavelength can be thought of as a measure of the spatial periodicity of fluid velocity; i.e., within a fixed dimension that is smaller than the wavelength, a longer wavelength results in smaller spatial variation of fluid velocity. However, velocity differences that occur within the fluid directly determine the magnitude of dissipation; i.e., at the same fluid viscosity smaller velocity differences result in lower friction and consequently less dissipation. On the other hand, when the critical dimension exceeds one-quarter of the wavelength, it becomes large enough to accommodate fluid with velocity differences in that dimension, leading to a significant increase in dissipation.

It is important to note that the fundamental frequency affects the master design plots (i.e., 5 MHz’s and 10 MHz’s plots are different), because these structures are not perfectly rigid and thus have dynamic response with respect to the fundamental frequency. Figures S17 and S18 show that the overall rigidity of the structure affects dissipation (for instance, factors such as height, thickness, and Young’s modulus impact the overall rigidity). We speculate that if the structure were perfectly rigid, the fundamental frequency would not affect dissipation. Alternatively, if waves transmitted within the structure exhibited nearly non-dispersive characteristics (for instance, if the structure were a thicker solid rather than an irregular thin wall), the dissipation would not be affected by the fundamental frequency either. We did not model and analyze this scenario because we aimed to make our model closely reflect our experimental conditions. However, our experimental condition does not allow us to deposit a very thick metal layer on a conventional QCM, as this would lead to crystal overloading.

Dissipation is generally minimized with decreasing $H/{\lambda }_{{\rm {s}}}$ and with selecting a $W/{\lambda }_{{\rm {p}}}$ value outside a critical region (yellow highlights in Fig. 7). We attribute the notable increase in dissipation in the center region of the graphs in Fig. 7 to the maximization of the velocity difference in the fluid (due to a resonance in the liquid), which in turn leads to maximized friction. Practically, dissipation can be minimized by decreasing channel height *H* and selecting a channel width $W$ so as to move away from the high dissipation region. It is evident from Fig. 7 that the various modes of the µ-QCM employed in the experiment all reside within the low dissipation region.

This dimensional analysis also provides a route to rationally design other acoustic biosensors with minimized dissipation. For example, microfluidic channels similar to ours can also be built on SAW or FBAR sensors and be used to diminish dissipation either through pressure wave (${\lambda }_{{\rm {p}}}$) suppression or through shear wave (${\lambda }_{{\rm {s}}}$) suppression, as long as the design of the critical dimensions lies within the low dissipation region.

## Discussion

Low *Q*-factors of acoustic biosensors arise from dissipation through acoustic radiation and liquid friction. For the past decades, researchers have devoted significant effort to suppress acoustic radiation losses either by energy trapping or by switching from out-of-plane acoustic modes to in-plane acoustic modes. Nonetheless, the problem of low *Q*-factors resulting from liquid friction has still not been fully addressed. In this study, we built a µ-QCM as a model system to demonstrate that a pressure wave can be used to suppress liquid friction resulting from a shear evanescent wave. To this end, we developed the µ-QCM by attaching rigid microfluidic channels to the surface of a conventional QCM crystal. In this new sensing paradigm, the microfluidic channels, placed perpendicularly to the shearing direction of the quartz crystal, generate pressure waves in the liquid entrapped in the channels. The channel width ($W$) is chosen such that it is much narrower than the pressure wavelength ${\lambda }_{{\rm {p}}}$. In this case, no velocity variations occur in the liquid across each channel, which suppresses dissipation from liquid friction.

The extent of suppression of liquid friction is mainly limited by the width and the rigidity of the microfluidic channels. The first limitation arises when increasing the fundamental resonance frequency because then the channel width must be made smaller. The second limitation arises from the finite rigidity of the microfluidic channels, which also affects the suppression, as shown in Figs. S17 and S18. For this paper, we fabricated the microfluidic channels via a standard photolithographic process, subsequent e-beam evaporation (EVAP), and direct current (DC) magnetron sputtering of aluminum. This approach reaches its limitations when smaller channel dimensions are needed. Furthermore, the rigidity of the microfluidic channels could be further improved by switching from additive to subtractive manufacturing techniques.

## Conclusions

Here, we present a novel approach for improving dissipative losses, and hence the Q-factor, of acoustic gravimetric biosensors operating in a liquid environment. Specifically, we fabricated the μ-QCM by placing rigid microfluidic channels on top of conventional QCM sensors, using photolithographic approaches that are commonplace for the fabrication of microelectromechanical devices. The microfluidic channels are passively filled with probe liquid by capillary actions. Compared with conventional QCM sensors, the experimental characterization of our μ-QCM sensors revealed a more than 10-fold improvement in the dissipation shift when operating in a liquid environment. This indicates the elimination of essentially all liquid-induced dissipation. Furthermore, we found that the μ-QCM is sensitive to changes in liquid mass (density) only, while the conventional QCM is influenced by a convolution of density and viscosity.

To gain a deeper understanding of the physics underlying the operation of the µ-QCM, we employed finite element analysis (FEA). We constructed our simulation models by using a hierarchical building process and we validated these models against experimentally obtained results. Our FEA, in combination with dimensional analysis, revealed two key factors that affect device performance: (1) the ratio of the channel width to the pressure wavelength ($W/{\lambda }_{{\rm {p}}}$) and (2) the ratio of the channel height to the shear evanescent wavelength ($H/{\lambda }_{{\rm {s}}}$). By mapping the dissipation response as a function of these two dimensionless ratios, we generated a contour plot that can be used to guide the design and fabrication of other acoustic biosensors.

The μ-QCM offers not only a 10-fold decreased dissipation shift due to liquid loading but also direct determination of changes in liquid density (mass), minimized sample volume requirements, and less stringent temperature control. These combined advantages make the μ-QCM potentially attractive for various biosensing applications, especially for point-of-care (POC) settings.

## Methods

### Device fabrication process

To fabricate a µ-QCM with rigid microfluidic channels of precise dimensions we chose a process that differs from the commonly used methods for fabricating microfluidic devices, such as soft lithography, hot embossing, laser ablation, or micro-milling. Instead, we used a traditional microfabrication process that is widely employed in the manufacture of microelectromechanical devices^50^.

**Fig. 8:**
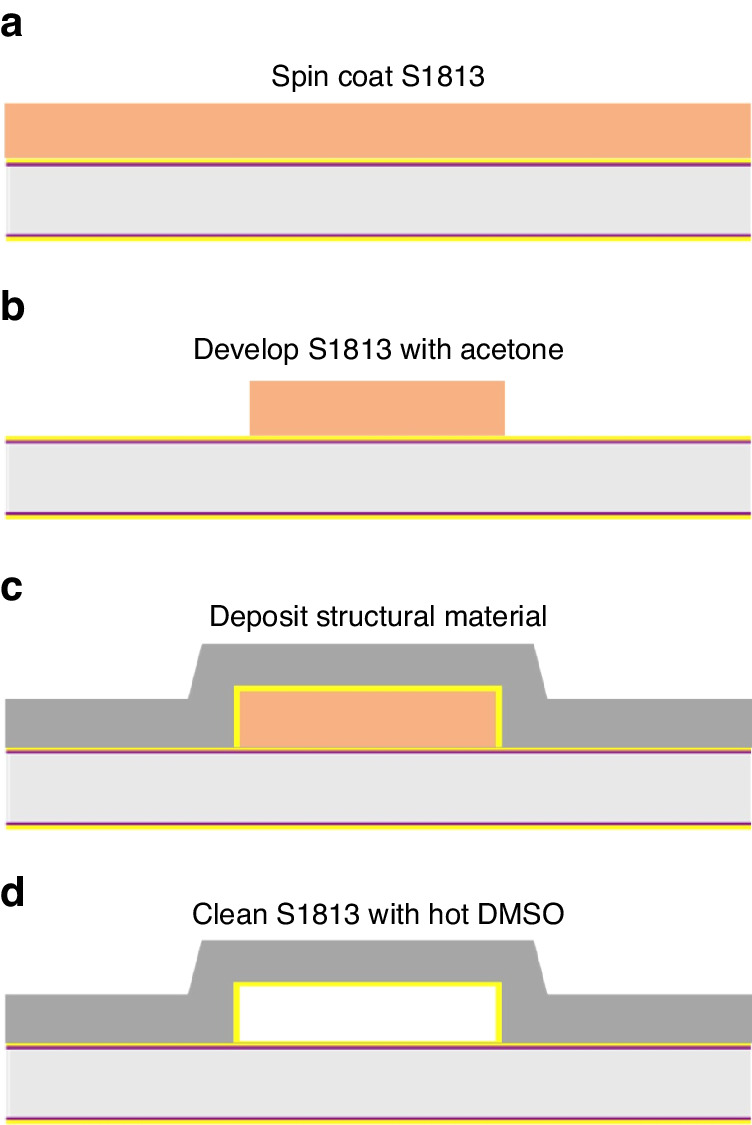
Single device fabrication process. A widely employed MEMS device fabrication process was used to fabricate the µ-QCM. Main steps include spin-coating of photoresist (S1813), photolithography, DC sputtering and lift-off with DMSO

Our fabrication process consists of three main steps: (i) photolithography, (ii) thin film deposition, and (iii) lift-off (Fig. 8). Initially, a 2 µm-thick layer of S1813 photoresist was spin-coated onto a 4-inch AT-cut quartz wafer that already had an array of gold electrodes on its bottom and top surface. These electrodes were deposited by electron-beam physical vapor deposition (EBPVD). The wafer was then micropatterned by photolithography, using a borosilicate glass photomask. After exposure, the resist was developed in MF-319 developer for 60 s with gentle agitation. The wafer was subsequently cleaned with DI water, dried in a stream of N_2_, and then cleaned for 1 min in an O_2_ plasma. The fluidic channels were created by DC sputter, depositing thin films of 40 nm gold, 40 nm titanium, and 2 µm aluminum on the wafer. After deposition, the wafer was diced into 32 square devices, which still had their microchannels filled with S1813 resist. The liquid in- and outlet on each device was then scratched open using pointed tweezers. To remove the residual photoresist in the microchannels, the devices were immersed in DMSO at 60 °C for 4 days, followed by sequential cleaning in acetone and isopropyl alcohol for 1 day, respectively.

### Device characterization

We used a commercially available QCM analyzer with dissipation monitoring (Q-Sense, Biolin Scientific) to evaluate the response of µ-QCM and conventional QCM sensors. This analyzer allows for monitoring of the resonance frequency shift with selected overtones and the corresponding dissipation shifts as a function of time.

The sample liquids employed in our study were DI water and a range of water-ethanol mixtures (10–30 wt% ethanol). Prior to admitting any sample liquid, baseline data for the resonance frequency with overtones and their corresponding dissipation were recorded. Subsequently, 5 µL of sample liquid was added to the inlet channel of the µ-QCM via pipette, where liquid was entrained into the microchannels by capillary action. During a measurement cycle (Fig. S21) we ensured that a sufficient volume of sample liquid remained positioned on the inlet port to avoid evaporation, which can lead to entrainment of air bubbles into the channels, thus impeding capillary action and channel filling.

### Finite element analysis (FEA)

**Fig. 9:**
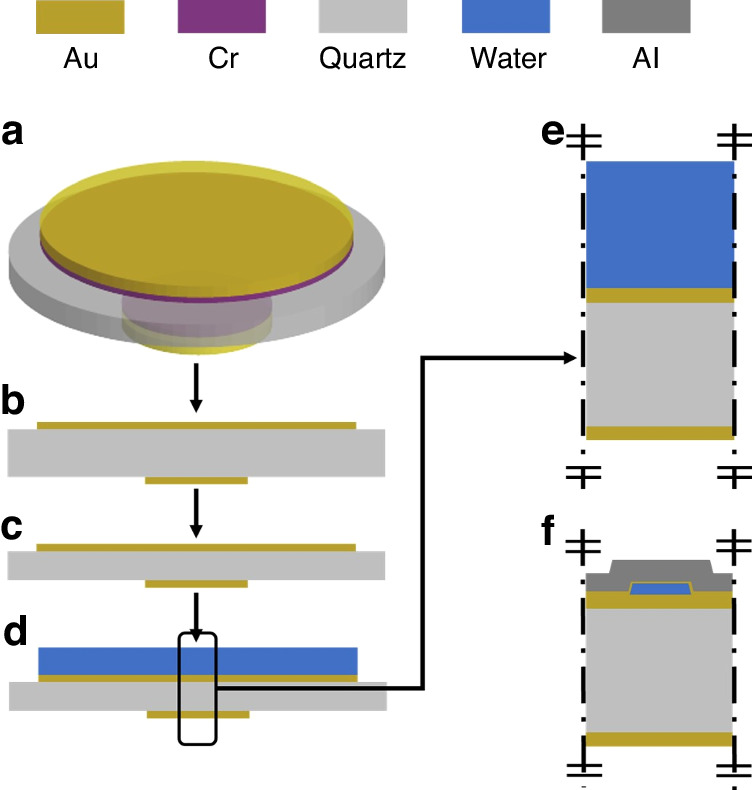
Hierarchical FEA model building process. **a** 3D conventional QCM in vacuum. **b** and **c** 2D conventional QCM in a vacuum with different crystal thicknesses. **d** 2D conventional QCM with liquids. **e** 2D center model for conventional QCM. **f** 2D center model for µ-QCM

To fully understand the physics that gives rise to the observed non-dissipative behavior in µ-QCM we conducted a thorough FEA. Specifically, we used the acoustic module of COMSOL Multiphysics for our simulations, which consist of a coupled system of thermo-viscous acoustics, solid mechanics, and piezoelectricity. Our final models for both the conventional QCM and the µ-QCM were derived from a hierarchical model-building process (3-D to simplified 2-D), shown in Fig. 9, and detailed further in the SI.

The 2-D simplification in the final model was imperative to conserve computational resources, because the dimensions of the microfluidic channels (2 µm in height and 10 µm in width) are significantly smaller than the smallest dimension (330 µm in thickness) of the underlying quartz crystal, and a dense mesh configuration is required to accurately capture the behavior of wave propagation within the channels. This results in a substantial number of elements or degrees of freedom (DOF) in the simulations. Consequently, we made several assumptions to reduce the size of the model:

1. Any variations along the length of the channels are assumed to be negligible. This is supported by the high aspect ratio of channel length (5 mm) to channel width (10 µm). This enables a 2-D representation of the device using a center cut and a plain strain simplification.
2. The presence of electrodes, gold, and titanium layers is assumed to be insignificant to wave propagation. This is rationalized by the negligibly small thickness of these layers (~40 nm) compared to the thickness of the aluminum layer of the channels (2 µm).
3. Any variations perpendicular to the channels are also assumed to be negligible. This is rationalized by the large ratio of crystal diameter (1 cm) to channel width (10 µm) and the small distance (20 µm) between the channels. Since the channels appear in a repeating pattern with a distance of 20 µm, we applied periodic boundary conditions in the model.

The final 2-D model of the µ-QCM thus contains only a single microfluidic channel situated on a quartz crystal.

To extract data from our simulations, we conducted a frequency sweep for the total electrical energy, which serves as an indicator of the total stored electrical energy in the system. The peak in the frequency sweep corresponds to a resonance, and the ratio of its full width at half maximum (FWHM) to its resonance frequency yields the Q-factor^51^. We validated our final FEA models through comparison with experimental data (see results section). The models were also verified through a p-extension (different polynomial degrees) mesh convergence study. More details can be found in the SI.

To gain a comprehensive understanding of the underlying physics, we perturbed several key parameters in the simulations, including the channel height, the channel width, the liquid density, the liquid viscosity, the liquid bulk compressive modulus, and the crystal thickness.

### Dimensional analysis

To extend our findings from µ-QCM to other acoustic biosensors, we conducted a dimensional analysis. Specifically, we used the Buckingham Pi Theorem for the analysis, using the critical parameters identified through our simulations including the fundamental resonance frequency ${f}_{0}$,the *Q*-factor $Q$, the channel height $H$, the channel width $W$, the crystal thickness $t$, the liquid density $\rho$, the liquid viscosity $\mu$, and the liquid bulk compressive modulus $\kappa$.

Using the Buckingham-PI theorem^52,53^, we derived a dimensionless equation, which demonstrates that the *Q*-factor is dependent on several dimensionless terms, as shown below,

5 $${\varPi }_{1}={f}^{{\prime} }\left({\varPi }_{2},{\varPi }_{3},{\varPi }_{4},{\varPi }_{5}\right)$$

6 $$\,{\varPi }_{1}=Q$$

7 $${\varPi }_{2}=H/W$$

8 $${\varPi }_{3}=t/W$$

9 $${\varPi }_{4}=\kappa /\rho {W}^{2}{f}_{0}^{2}$$

10 $${\varPi }_{5}=\mu /\rho {W}^{2}{f}_{0}$$

Among the dimensionless terms, ${\Pi }_{4}$ and ${\Pi }_{5}$ are of particular interest. They can be further simplified using equations that describe the pressure and shear evanescent waves in liquid. Assuming that ${\Pi }_{2}$ is a constant, the final equations for these two terms are shown below.

11 $${\Pi }_{{\rm {p}}}=1/{\varPi }_{4}={\left(W/{\lambda }_{{\rm {p}}}\right)}^{2}$$

12 $${\Pi }_{{\rm {s}}}=1/{\varPi }_{5}={\left(H/{\lambda }_{{\rm {s}}}\right)}^{2}$$

In these equations, ${\lambda }_{{\rm {p}}}$ and ${\lambda }_{{\rm {s}}}$ are the characteristic wavelength for the pressure and the shear evanescent wave in liquid, respectively. The two terms are related to (Eq. (11)), the ratio of the channel width to the pressure wave wavelength, and (Eq. (12)), the ratio of the channel height to the shear penetration length.

To further illustrate the effect of these dimensionless terms on dissipation, we generated 2-D contour plots using our final FEA models, which provides a more general understanding of the interdependencies of channel dimensions, liquid properties and dissipation. These insights can be applied for the design of other acoustic biosensors.

### Supplementary information

Supplemental Material
