# Supplementary material for: Microfluidic QCM enables ultrahigh Q-factor: a new paradigm for in-liquid gravimetric sensing
Source: Microsyst Nanoeng. 2024 Aug 26;10:116. doi: 10.1038/s41378-024-00732-2 (PMC11347674; doi:10.1038/s41378-024-00732-2)
Supplement: Supplementary file 1 — Supplemental Material [file 41378_2024_732_MOESM1_ESM.docx]

Supplementary Information

**Microfluidic QCM enables ultrahigh Q-factor: a new paradigm for in-liquid gravimetric sensing**

Yicheng Zhao^1^, Zehra Parlak^2^, Wenjun Yu^2^, Daniel French^2^, Wilkins Aquino^3^ and Stefan Zauscher^1,2*^

^1^ Thomas Lord Department of Mechanical Engineering and Materials Science, Pratt School of Engineering, Duke University, Durham, NC 27708, USA

^2^ QATCH Technologies Inc., Durham, NC 27701, USA

^3^ Civil and Environmental Engineering, Pratt School of Engineering, Duke University, Durham, NC 27708, USA

* Corresponding author: Stefan Zauscher (zauscher@duke.edu)

**Tables**

*Table S1. Response of Conventional QCM to DI Water. The Q-factor dropped significantly upon introduction of DI water.*

| Mode Number | Q-factor  (No Water) | Normalized Frequency Shift | Q-factor (with Water) |
| --- | --- | --- | --- |
| 1 | 17590 | 791 | 2697 |
| 3 | 68465 | 450 | 5108 |
| 5 | 67349 | 348 | 6353 |
| 7 | 18508 | 252 | 5778 |

*Table S2. Response of the µ-QCM to DI water. The Q-factor remains relatively unchanged upon introduction of DI water.*

| Mode Number | Q-factor  (No Water) | Normalized Frequency Shift | Q-factor (with Water) |
| --- | --- | --- | --- |
| 1 | 20576 | 1124 | 13159 |
| 3 | 62771 | 1444 | 30730 |
| 5 | 35996 | 1685 | 31083 |
| 7 | 16932 | 2036 | 14787 |

*Table S3. Material properties of the QCM’s for FE models.*

| Property | Units | Value |
| --- | --- | --- |
| Gold Density | (kg/m^3^) | 18,500 |
| Quartz Density | (kg/m^3^) | 2,649 |
| Quartz Q-Factor | (kg/m^3^) | 100,000 |
| Gold Modulus | (MPa) | 92,050 |
| Gold Poisson Ratio |  | 0.42 |
| Gold Q-Factor |  | 10,000 |
| Quartz Elastic Matrix | (MPa) | $\left[ \begin{matrix} 86,740 & 267,150 & -8,250 & 0 & -3,660 & 0 \\ 267,150 & 102,830 & -7,420 & 0 & 9,920 & 0 \\ -8,250 & -7,420 & 129,770 & 0 & 5,700 & 0 \\ 0 & 0 & 0 & 68,810 & 0 & 2,530 \\ -3,660 & 9,920 & 5,700 & 0 & 38,610 & 0 \\ 0 & 0 & 0 & 2,530 & 0 & 29,010 \end{matrix} \right]$ |
| Quartz Piezoelectric Matrix | (C/m^2^) | $\left[ \begin{matrix} 0.1710 & 0 & 0 \\ -0.0187 & 0 & 0 \\ -0.1520 & 0 & 0 \\ 0 & -0.0671 & 0.0670 \\ 0.0670 & 0 & 0 \\ 0 & 0.0670 & -0.0950 \end{matrix} \right]$ |

*Table S4. Dimensions of the conventional QCM sensor used in our experiments.*

| Property | Units | Value |
| --- | --- | --- |
| Quartz Thickness | (mm) | 0.330 |
| Quartz Diameter | (mm) | 14 |
| Top Electrode Diameter | (mm) | 10 |
| Bottom Electrode Diameter | (mm) | 5 |

*Table S5. Material properties for butyl rubber and steel.*

| Property | Units | Value |
| --- | --- | --- |
| Steel Density | (kg/m^3^) | 7.900 |
| Steel Speed of Sound | (m/s) | 5,790 |
| Butyl Rubber Density | (kg/m^3^) | 1,070 |
| Butyl Rubber Speed of Sound | (m/s) | 1,830 |

*Table S6. Aluminum material properties used in our model.*

| Aluminum Property |  | Value |
| --- | --- | --- |
| Density | (kg/m^3^) | 2,700 |
| Young’s Modulus | GPa | 70 |
| Poisson Ratio |  | 0.35 |
| Quality Factor |  | 100,000 |

**Figures**

# 3D full model for conventional QCM with symmetric electrodes.


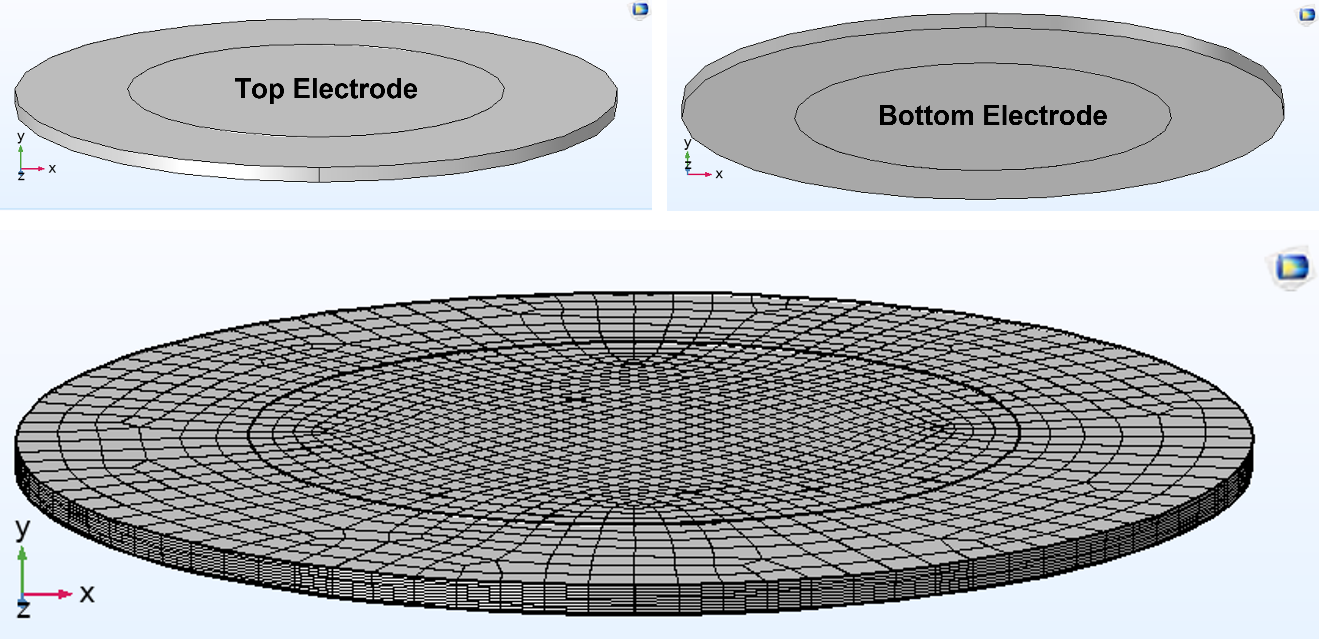


*Figure S1. 3D Symmetric Model. The quartz crystal disc has a diameter of 8 mm and a thickness of 185 um. The top and bottom electrodes are symmetric and have a diameter of 5 mm. Mesh configuration is shown at the bottom.*


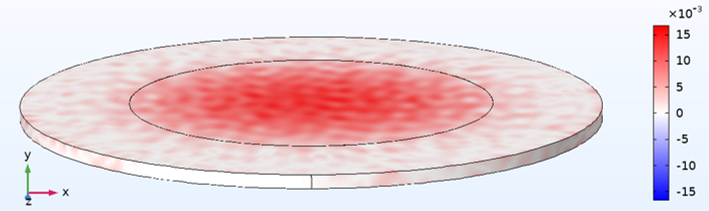


*Figure S2. Displacement Magnitude Mode Shape for the 3D Model of a conventional QCM. The primary direction of the vibration is in the x direction.*

# 2D full model for conventional QCM with symmetric electrodes.


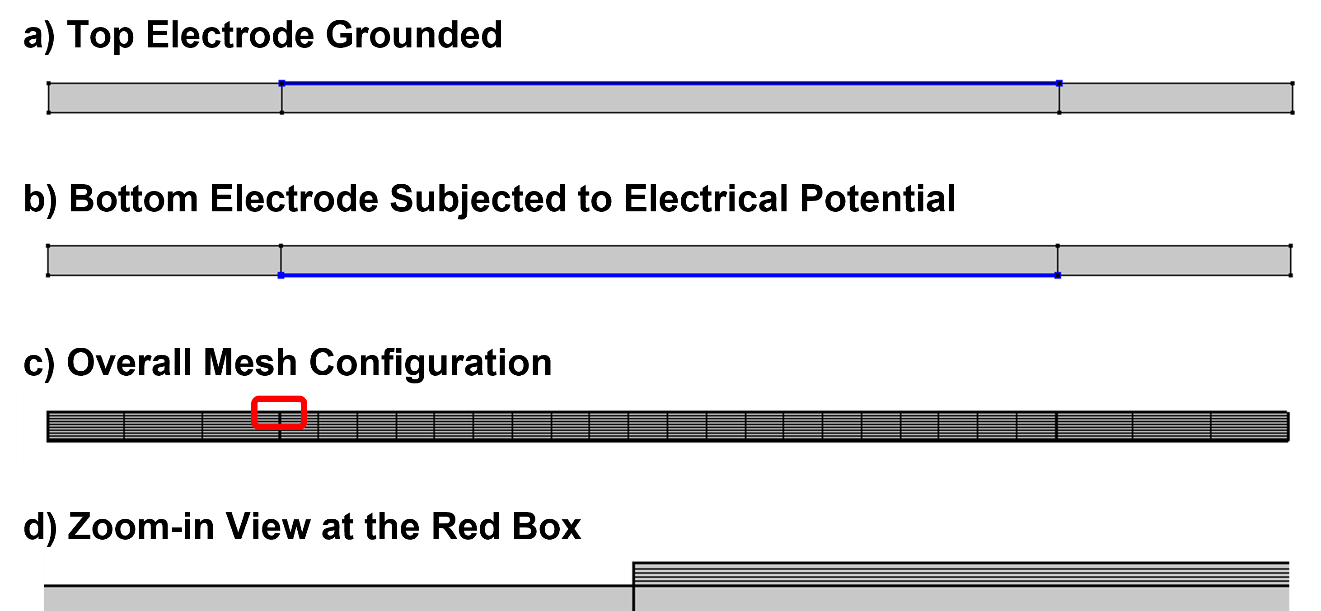


*Figure S3. Boundary Conditions and Mesh Configuration for the 2D Model. A) The top electrode is grounded. B) The bottom electrode is maintained at a voltage of 1V. c) The mesh is designed with a higher element density in the thickness direction. D) A particularly fine mesh is set up for the gold layer.*


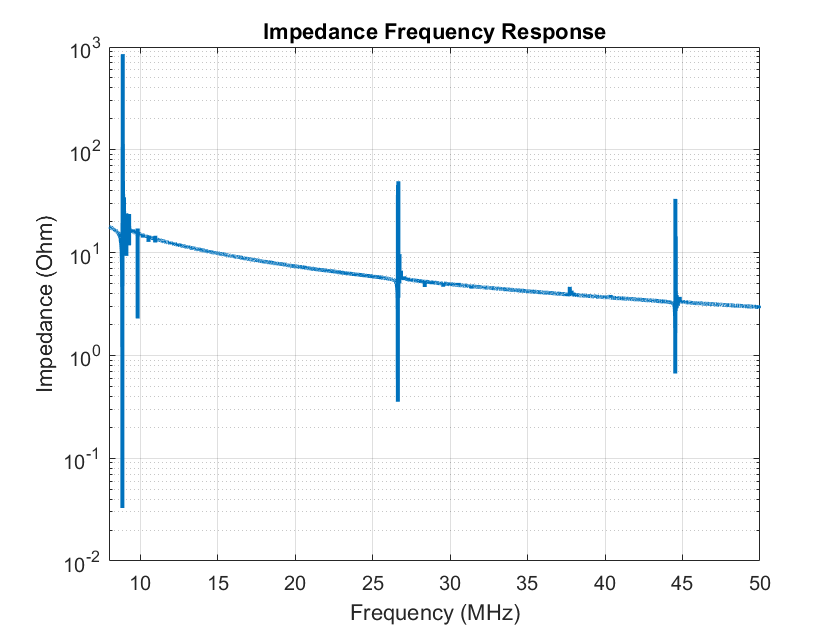


*Figure S4. Impedance Response for the 2D Model. The three large variations visible in the impedance response correspond to modes 1, 3 and 5.*


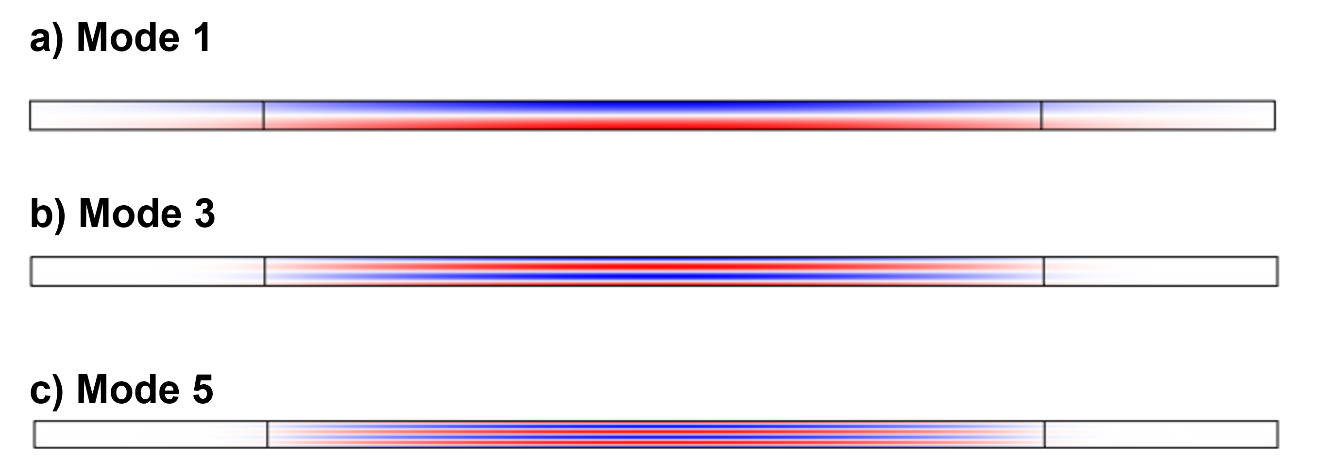


*Figure S5. Displacement Magnitude Mode Shapes for Mode 1, 3 and 5. The node number in each mode shape corresponds to modes 1, 3 and 5.*


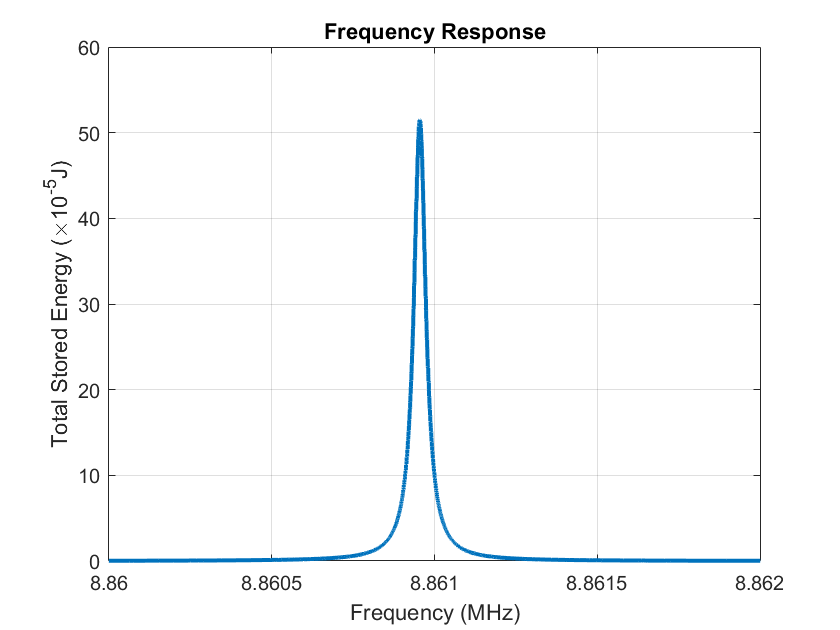


*Figure S6. Mode 1 Frequency Response for 2D Model. With finer frequency steps, 2D model shows smoother transition to its resonance peak.*

# 2D full model for conventional QCM with non-symmetric electrodes, representing actual QCM’s used in our experiments.


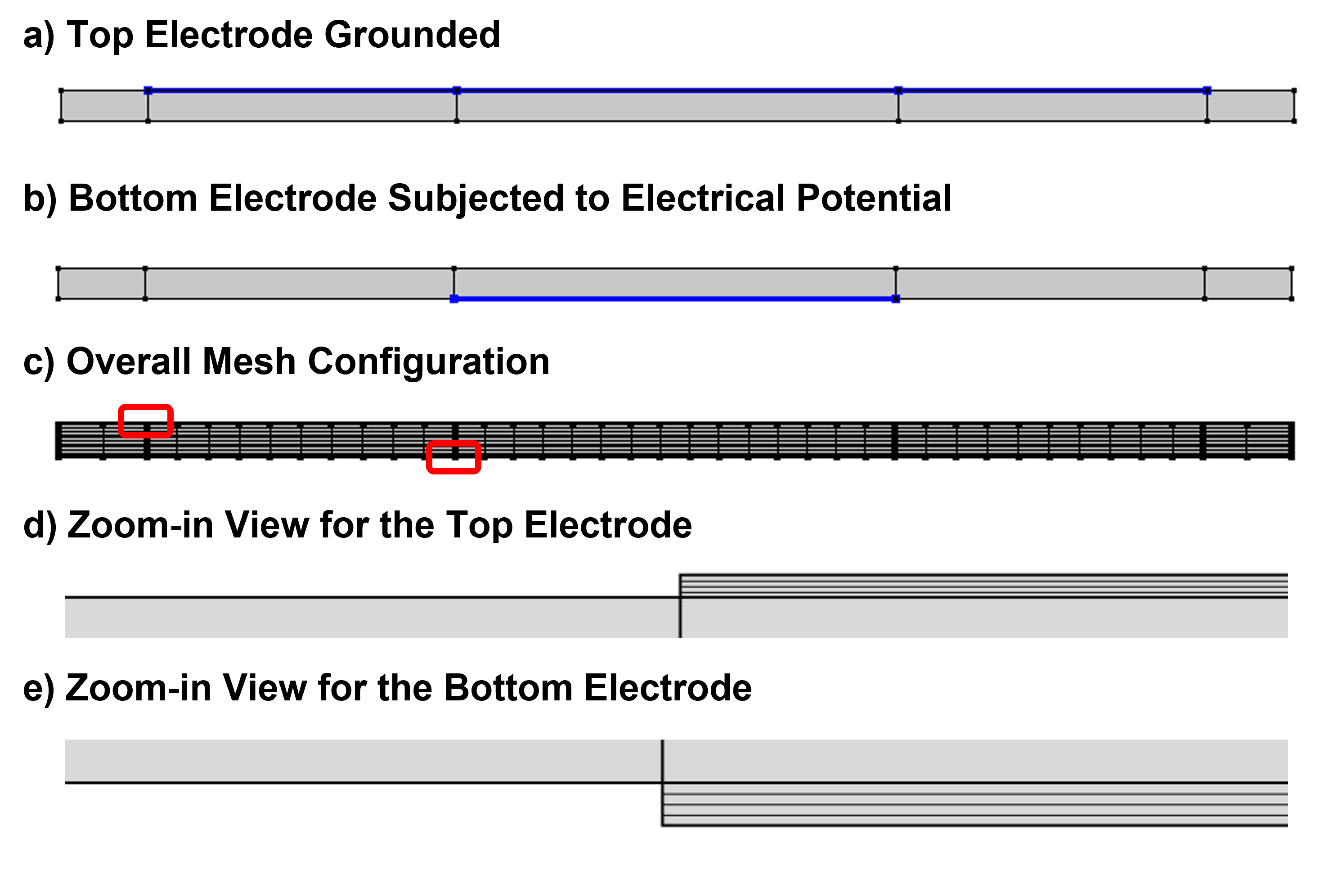


*Figure S7. Boundary Conditions and Mesh Configuration for Conventional QCM. This model resembles the conventional QCM we used in our experiments.*


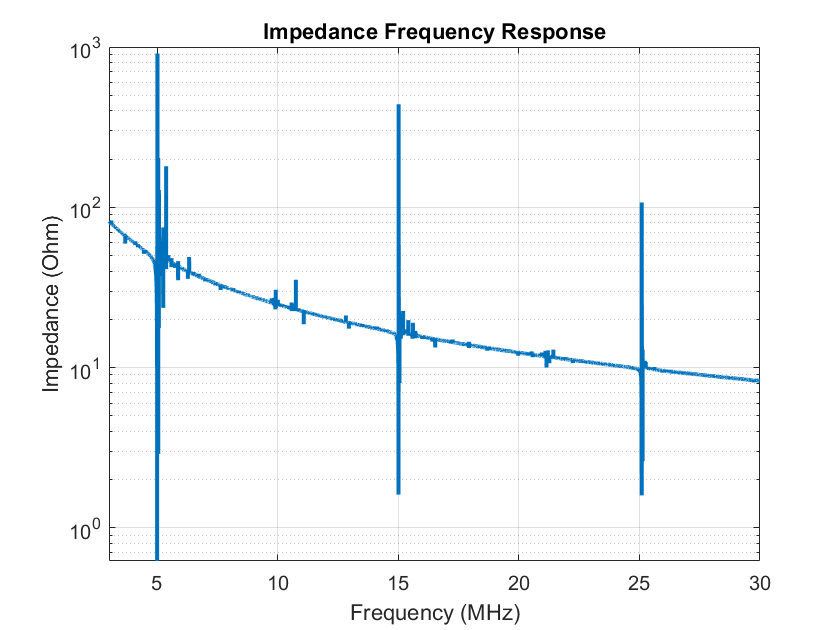


*Figure S8.* *Impedance Response for 2D Conventional QCM. Clear resonance behaviors at 5, 15 and 25 MHz are shown for modes 1, 3 and 5, respectively.*


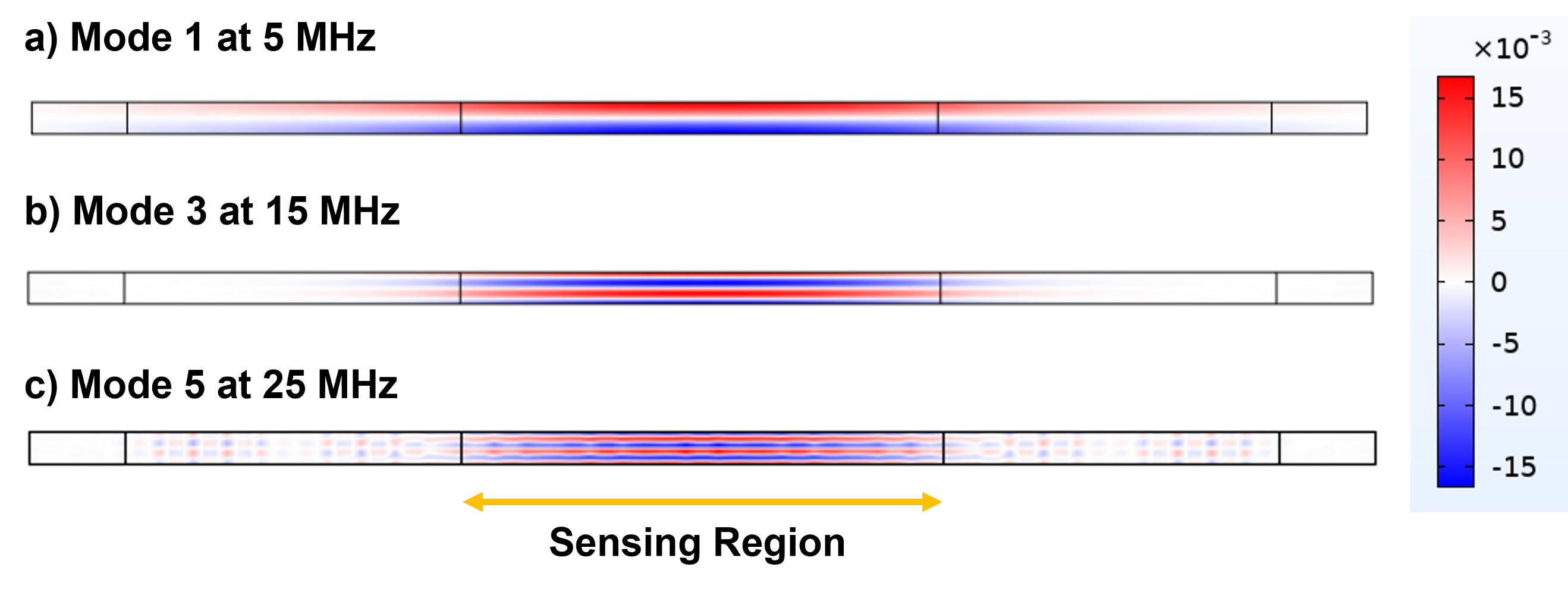


*Figure S9.* *Displacement Magnitude and Mode Shapes for Conventional QCM. The mode shapes are confined increasingly more to the sensing region with increasing mode number.*

# 2D full model for conventional QCM with sample liquids, representing actual experimental conditions for conventional QCM’s used in our study. Liquid properties used are those for water @ 25°C.


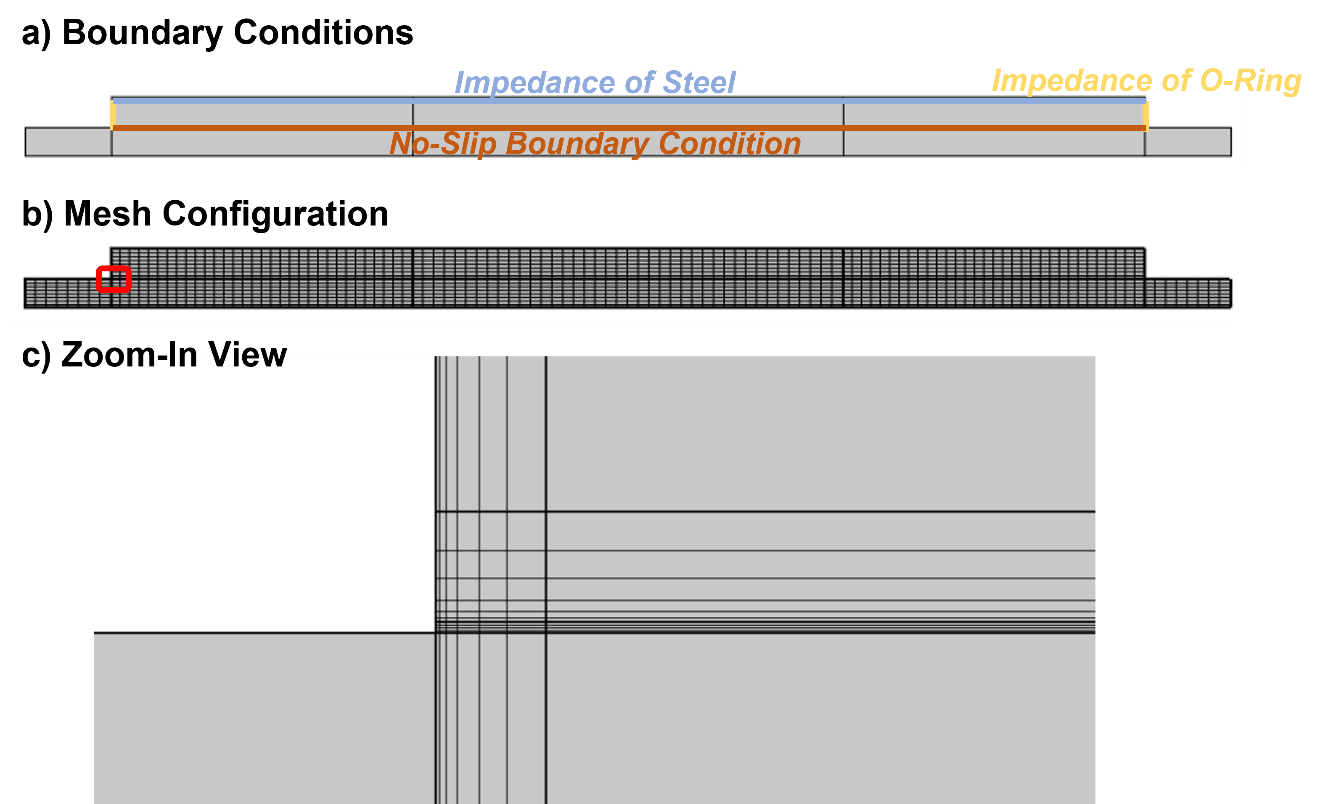


*Figure S10.* *Boundary Conditions and Mesh Configuration for Conventional QCM operating in Liquid. a) Schematic showing the updated boundary conditions. b) Schematic showing the mesh configuration, and c) zoom-in view of the red box showing the details of the mesh.*


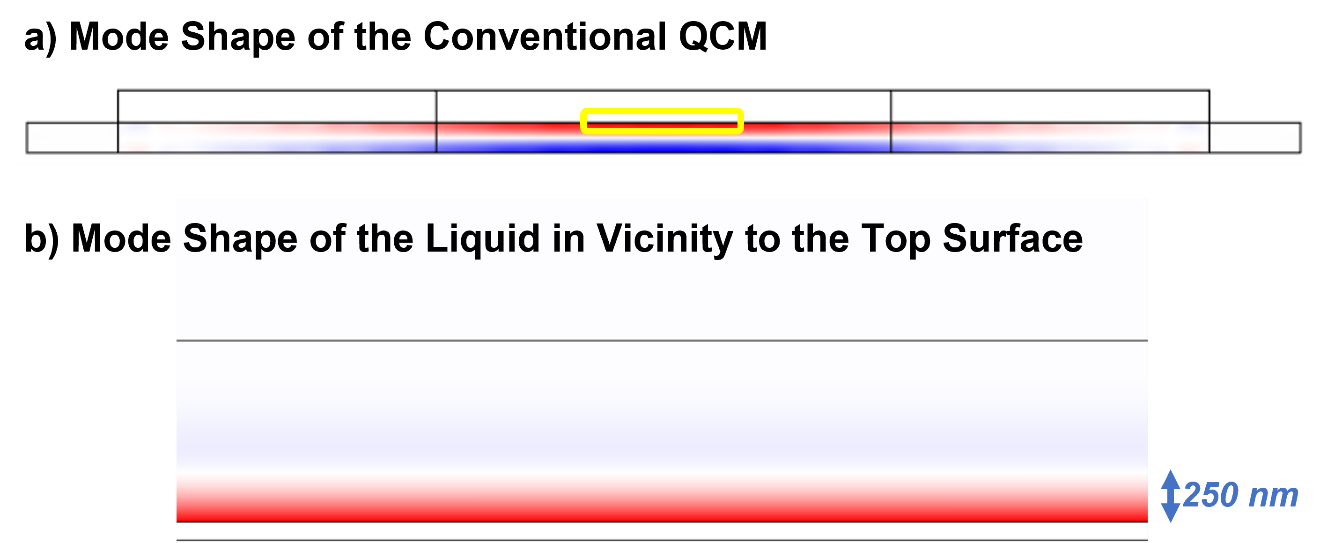


*Figure S11.* *Shear Evanescent Wave in 2D Simulation. a) shows mode shape of the conventional QCM b) shows the evanescent wave behavior of liquid in vicinity of the top electrode (zoom-in at the yellow box). Penetration length is measured to be 250 nm, which agrees with that calculated from acoustic theory.*

# 2D center model for conventional QCM with sample liquids, representing actual experimental conditions for conventional QCM’s used in our study. Liquid properties used are those for water @ 25°C.


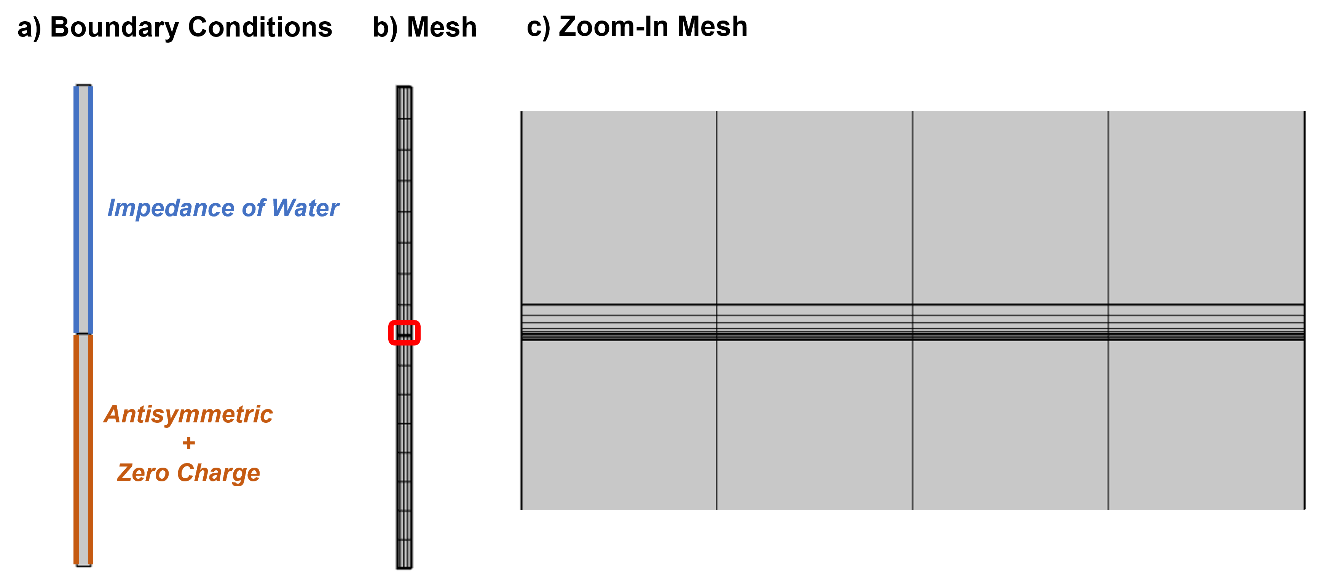


*Figure S12.* *2D Center Conventional QCM Model Configuration. We chose the boundary conditions as shown in a) to model the conventional QCM with a center repeating unit.*


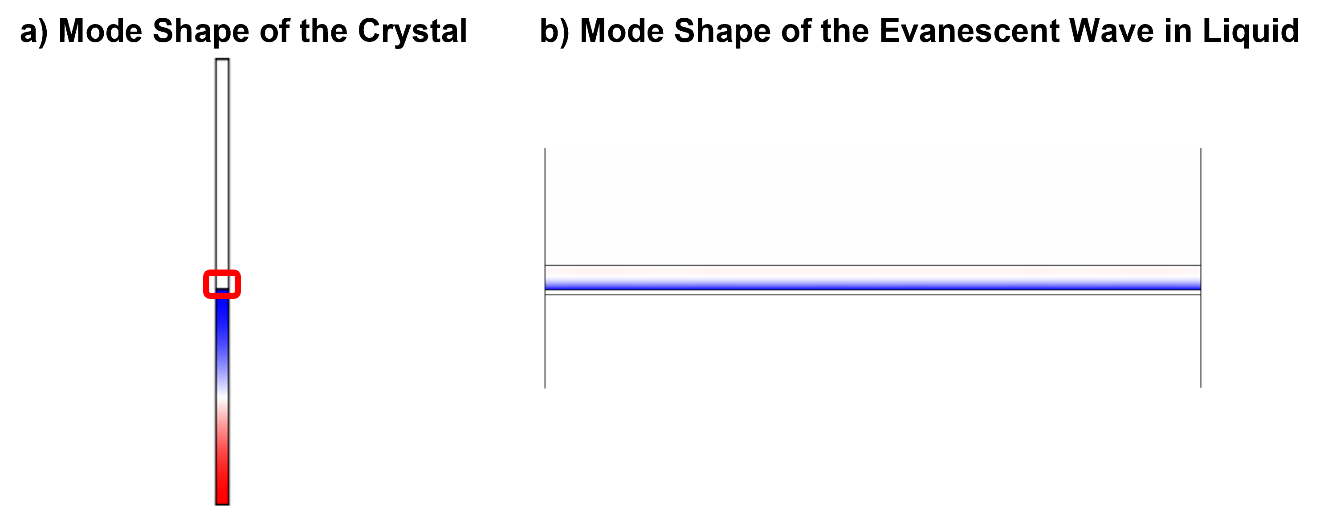


*Figure S13.* *2D Center Conventional QCM Mode Shape. a) Simulation showing the mode shape of the vibrating crystal. b) Simulation showing the mode shape of the liquid in vicinity of the top surface of the conventional QCM.*

# 2D full model for µ-QCM with sample liquids, representing actual experimental conditions for µ-QCM used in our study. Liquid properties used are those for water @ 25°C.


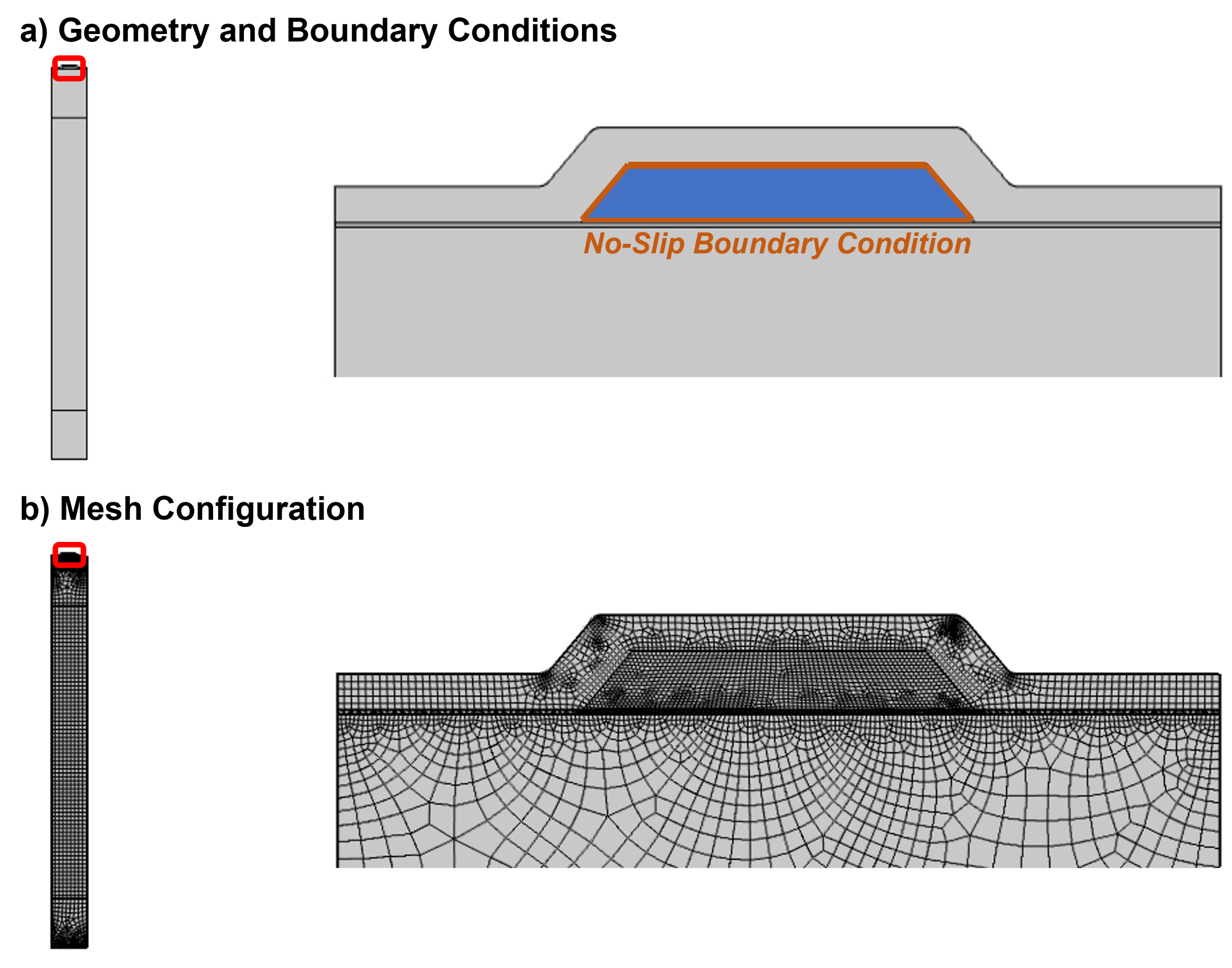


*Figure S14.* *2D µ-QCM Model Configuration. We use a repeating unit of a microfluidic channel to represent the µ-QCM. Models of the a) geometry and b) mesh configuration, with zoom-in views of the red boxes shown on the right.*

# Results from the mesh convergence study for the 2D center models.


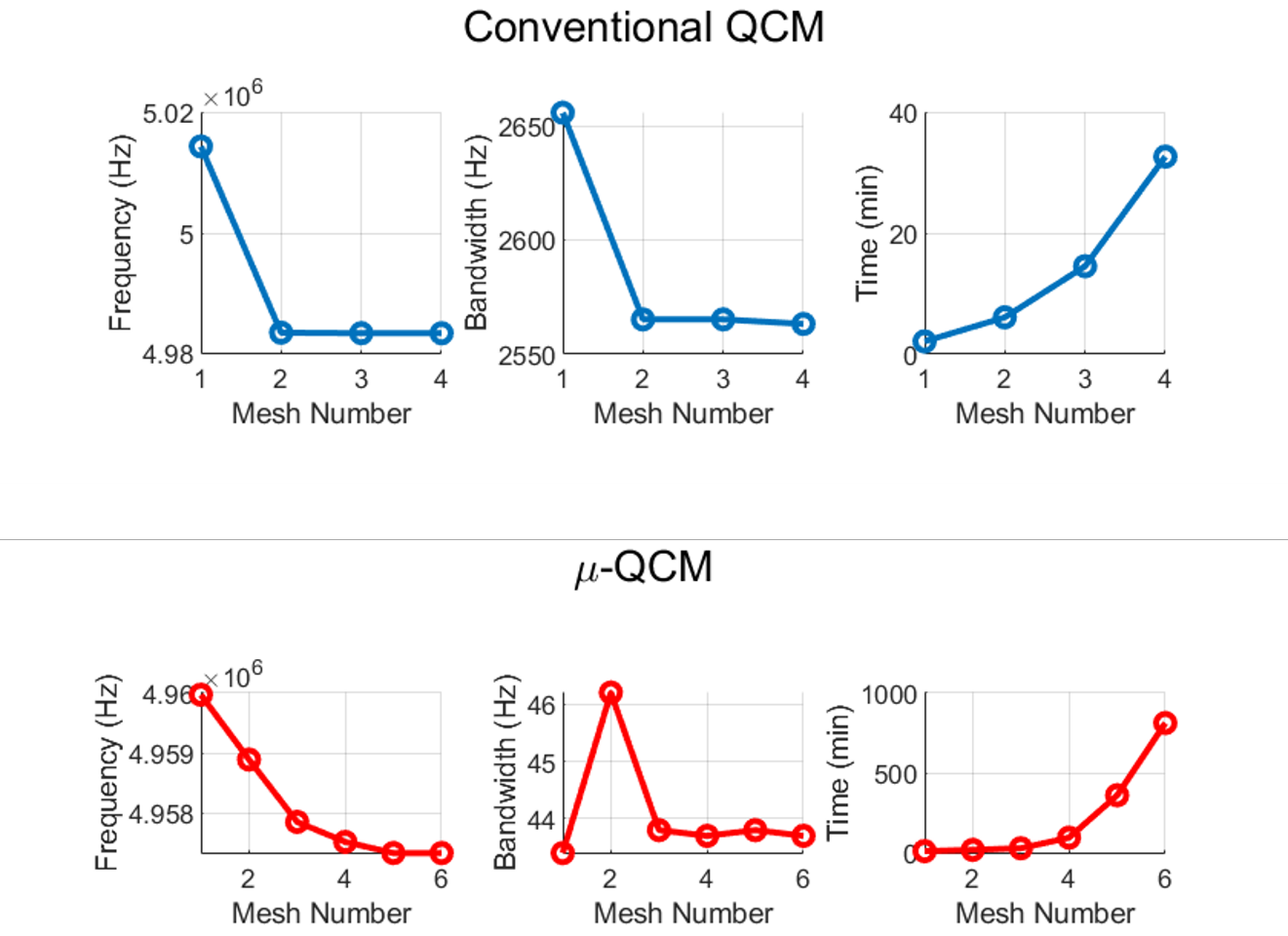


*Figure S15.* *Mesh Convergence. Top and bottom sections show mesh convergence for conventional QCM and the µ-QCM, respectively. An increasing mesh number indicates increasing DOFs either from p-extension or h-extension.*

# Results by changing density ($\rho$) and viscosity ($\mu$) for conventional QCM and µ-QCM models respectively. Initial liquid properties used are those for water @ 25°C.


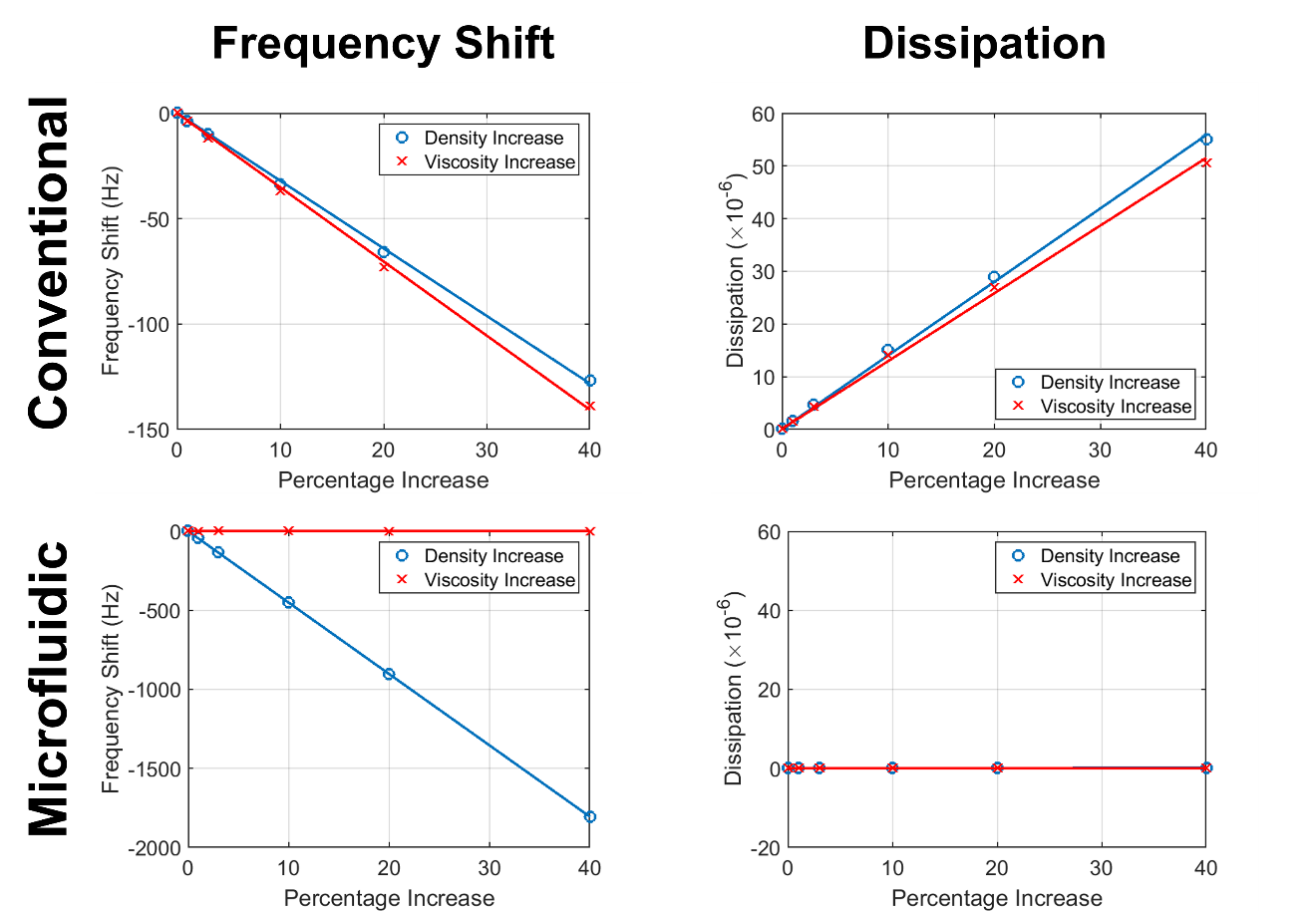


*Figure S16.* *FEA Results of Density and Viscosity Effects. The conventional QCM is sensitive to changes in both ρ and μ, while the µ-QCM is only sensitive to changes in ρ.*

# The overall rigidity of the microfluidic channels also affects dissipation. Initial material properties of the structure (Aluminum) are shown in Table. S6. Liquid properties used are those for water @ 25°C.


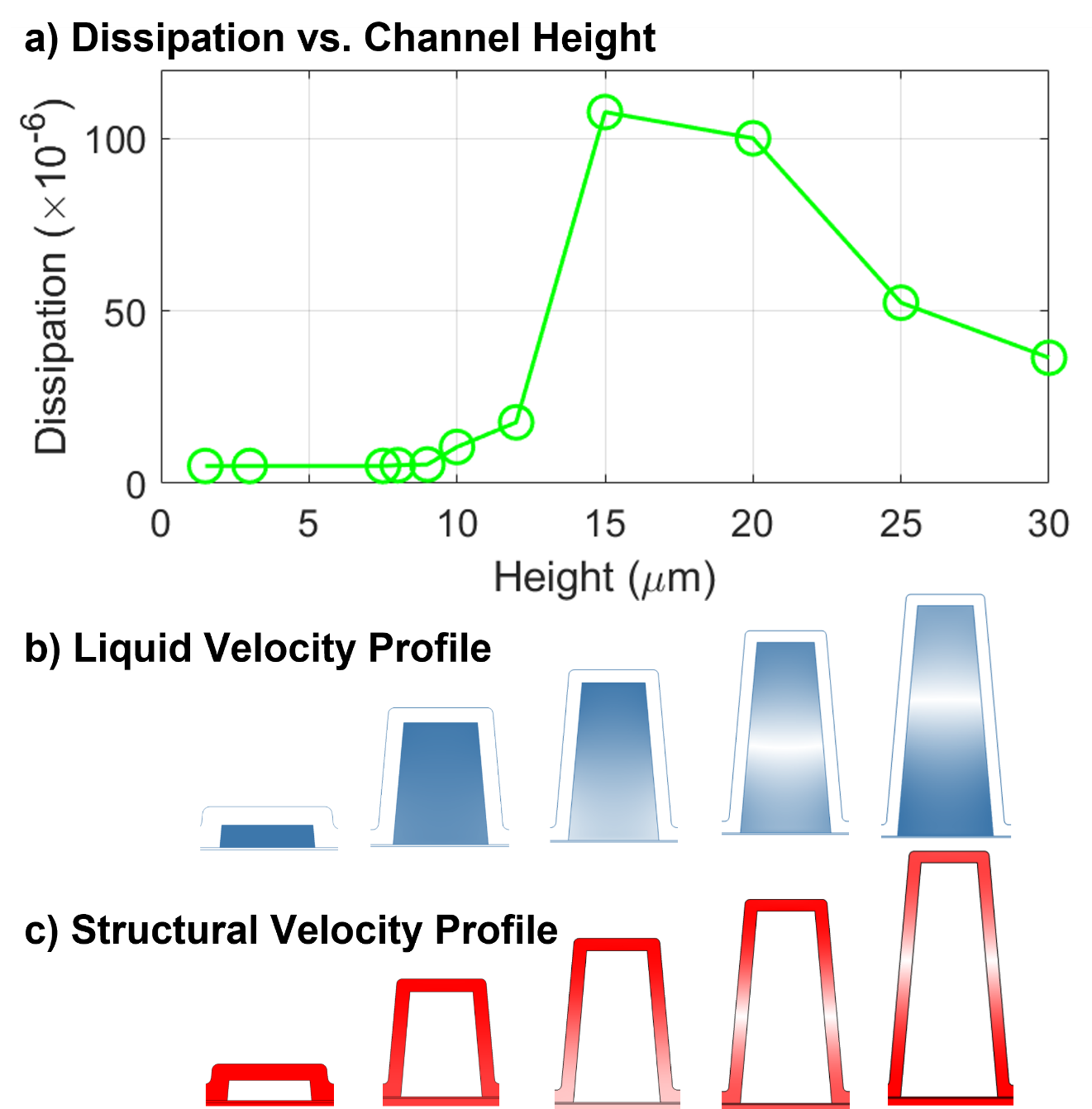


*Figure S17.* *Channel Height Analysis. Dissipation is minimized at channel heights lower than 10 μm but maximized at a channel height of 15 μm, for the fundamental resonance frequency of 5 MHz.*


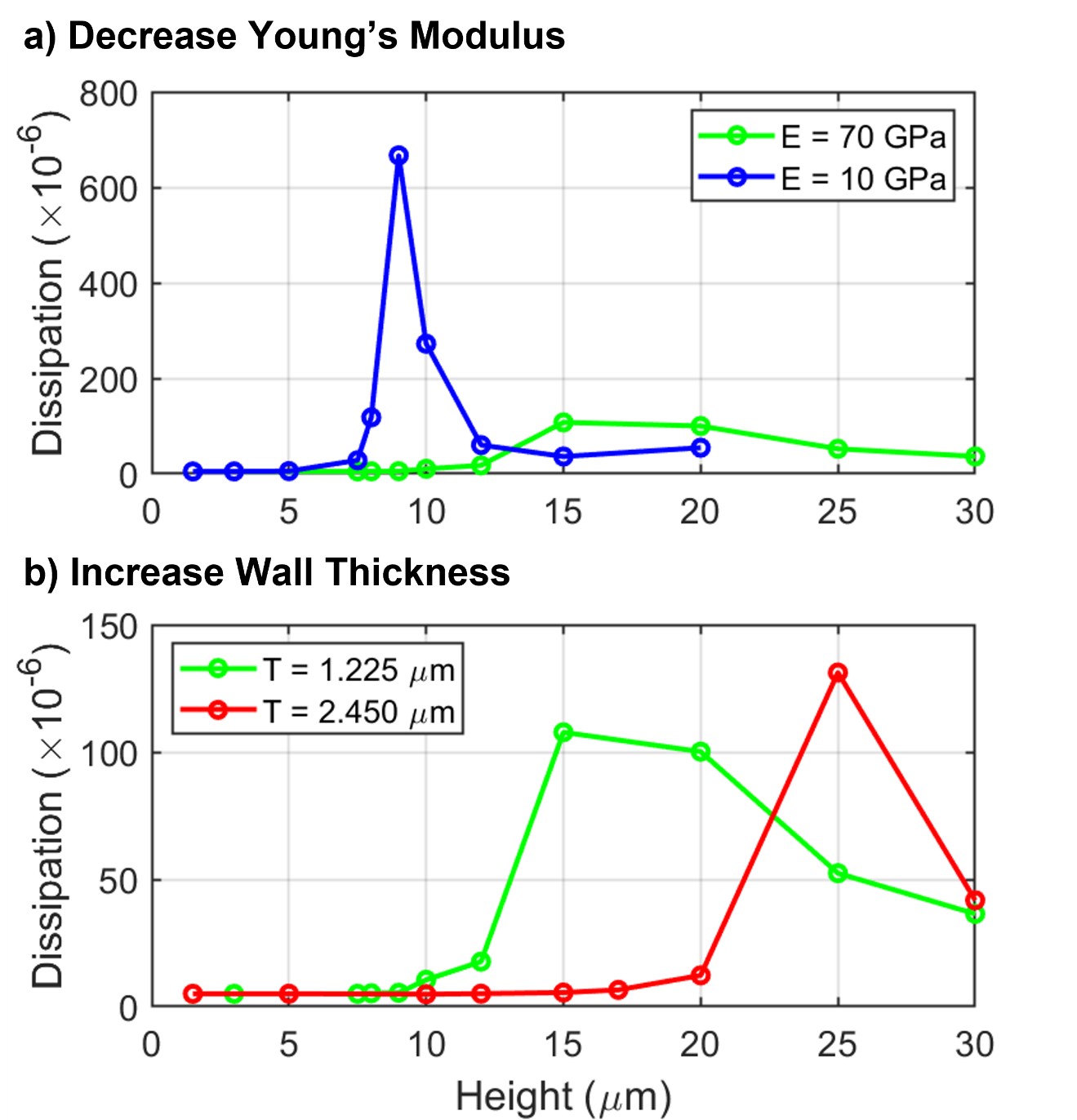


*Figure S18.* *Modulus and Thickness Effects. Green lines correspond to the same data, which is already shown in the previous section, while blue line corresponds to lower Young’s modulus and red line corresponds to thicker side wall.*

# Several microfluidic designs are tested to ensure bubble-free channels.


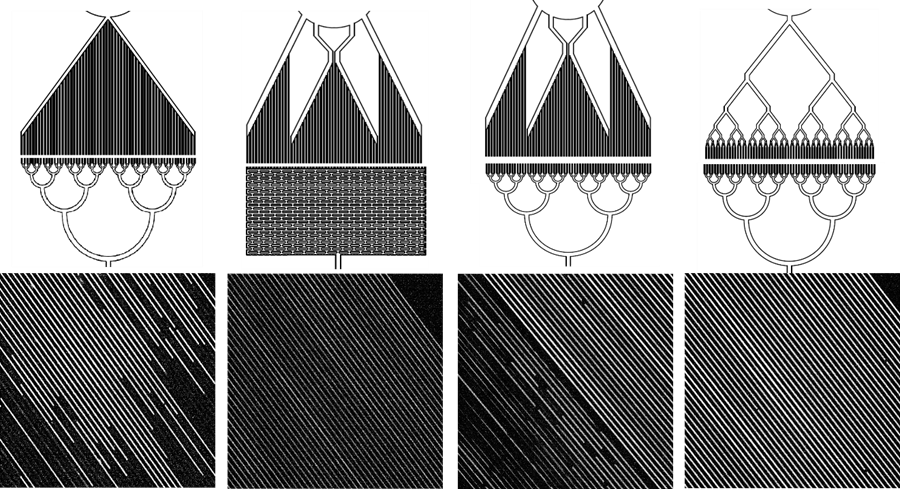


*Figure S19. Flow System Characterization. 2nd and 4th design combinations show best filling results, and the 2nd result is more consistent.*

# Lower modes are more sensitive to the outer edge due to the fact that mode shapes are more confined at higher modes.

*
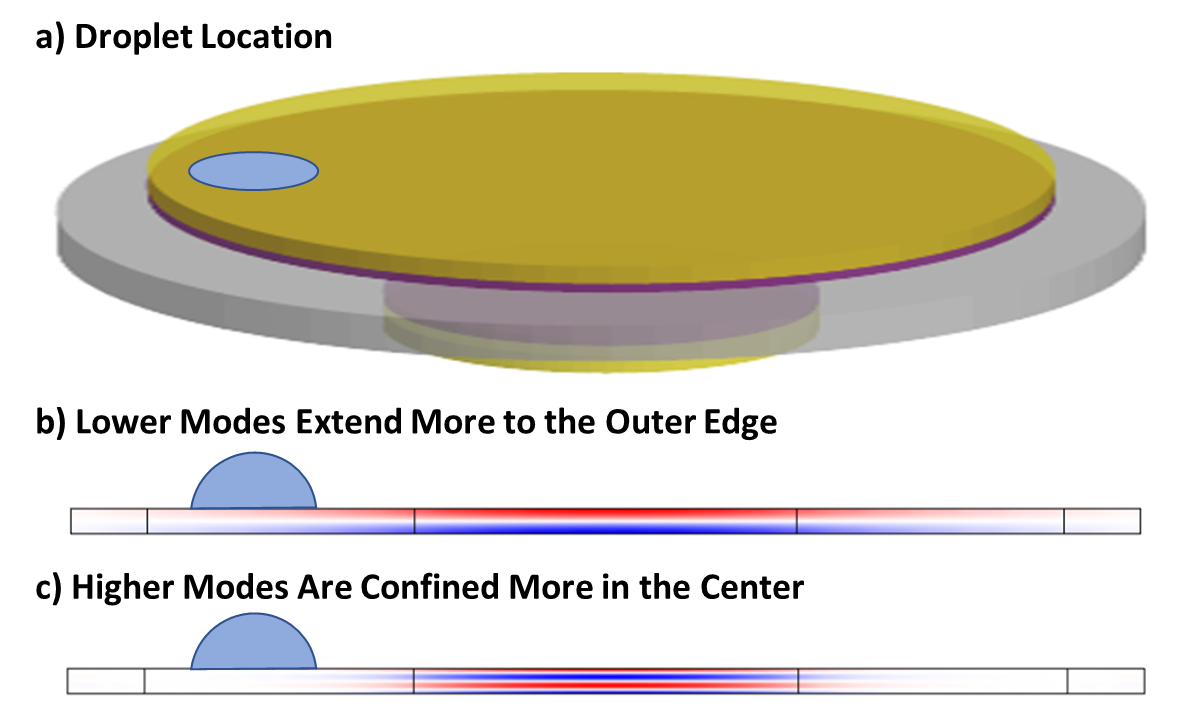
*

*Figure S20. Lower Modes Are More Sensitive to the Outer Edge. a) A small sample droplet is added to the inlet on the edge of the top electrode. b) Lower modes extend to the outer edge, and are thus sensitive to the droplet. c) Higher modes are more confined to the center region, and less sensitive to loading at the outer edge.*

# Experimental procedure for filling all microfluidic channels and ensuring bubble-free condition.

*
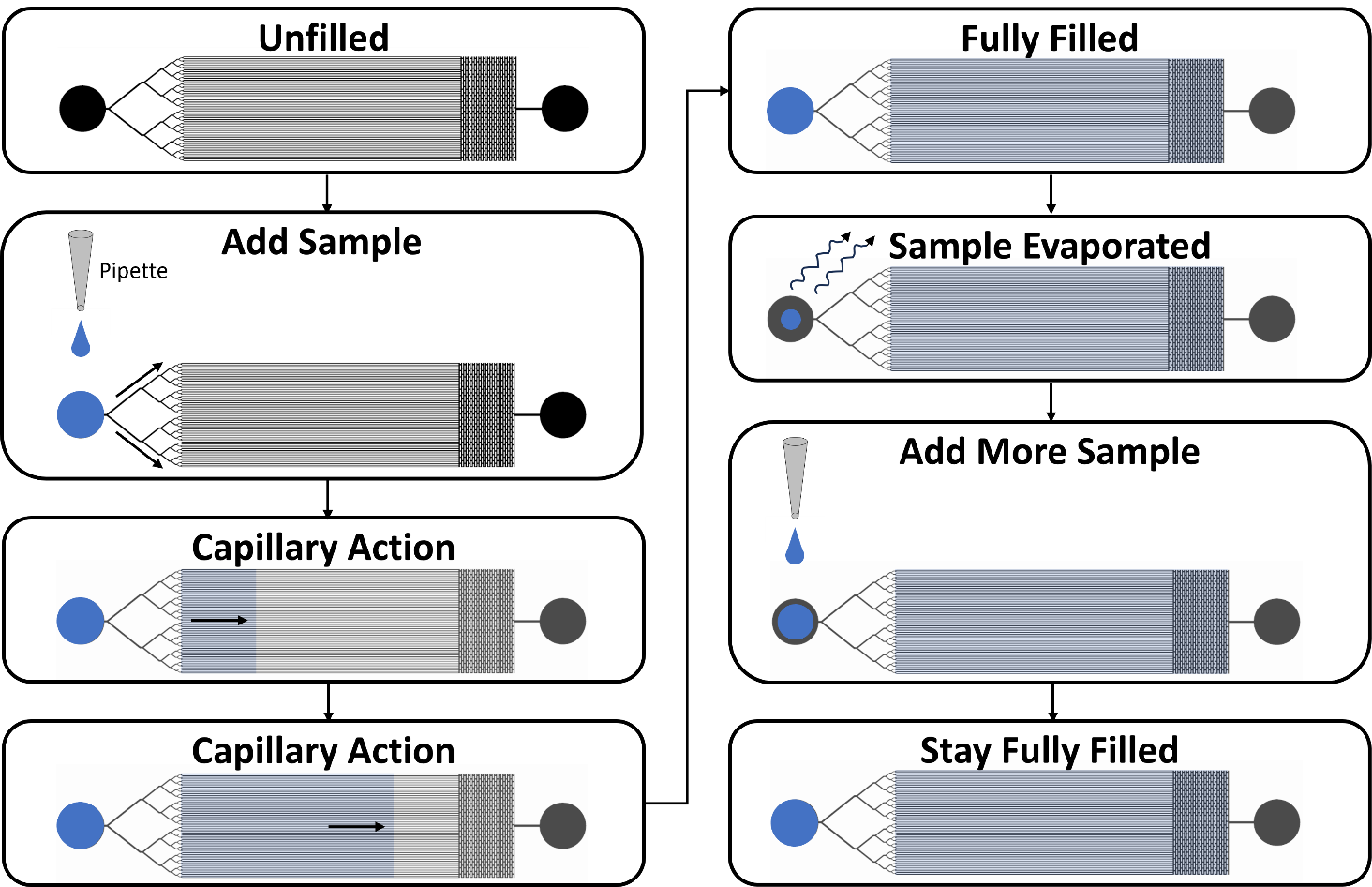
*

*Figure S21. Samples Are Filled With Capillary Actions. First, a sample droplet is added to the inlet through a pipette. Then, the sample fills all channels by capillary actions. During the measurement, the sample droplet at the inlet might evaporate. Thus, more sample is added to the inlet to prevent bubbles, which might form due to the evaporation.*

# Parallel oriented channels (with respect to the shearing direction) show similar results as conventional QCM (i.e., high dissipation).

*
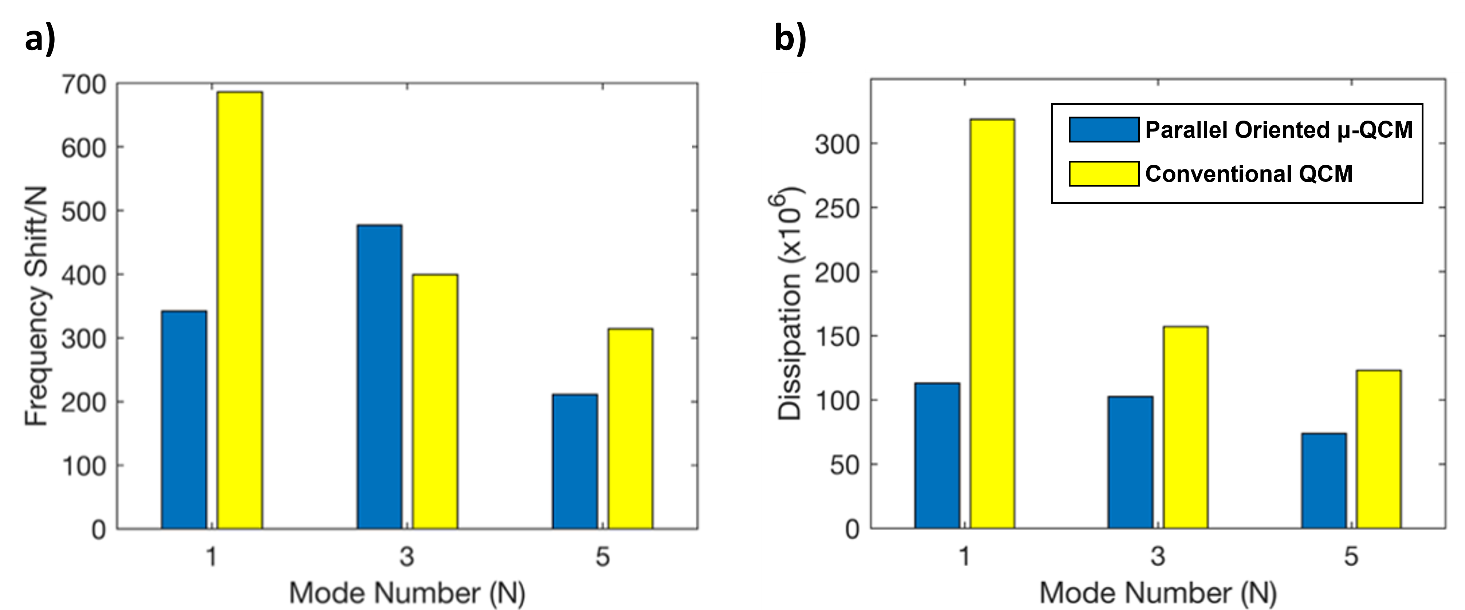
*

*Figure S22. Parallel Oriented Channels’ Response to DI Water Loading. In general, both the parallel oriented µ-QCM and the conventional QCM exhibit similar responses to DI water, displaying higher dissipation compared to perpendicular oriented µ-QCM.*
